# MODER2: first-order Markov modeling and discovery of monomeric and dimeric binding motifs

**DOI:** 10.1093/bioinformatics/btaa045

**Published:** 2020-01-30

**Authors:** Jarkko Toivonen, Pratyush K Das, Jussi Taipale, Esko Ukkonen

**Affiliations:** b1 Department of Computer Science, University of Helsinki, Helsinki FI-00014, Finland; b2 Applied Tumor Genomics, Research Programs Unit, University of Helsinki, Helsinki FI-00014, Finland; b3 Department of Biochemistry, University of Cambridge, CB2 1GA Cambridge, UK; b4 Division of Functional Genomics and Systems Biology, Department of Medical Biochemistry and Biophysics, SE 141 83 Stockholm, Sweden; b5 Department of Biosciences and Nutrition, Karolinska Institutet, SE 141 83 Stockholm, Sweden; b6 Genome-Scale Biology Program, University of Helsinki, Helsinki FI-00014, Finland

## Abstract

**Motivation:**

Position-specific probability matrices (PPMs, also called position-specific weight matrices) have been the dominating model for transcription factor (TF)-binding motifs in DNA. There is, however, increasing recent evidence of better performance of higher order models such as Markov models of order one, also called adjacent dinucleotide matrices (ADMs). ADMs can model dependencies between adjacent nucleotides, unlike PPMs. A modeling technique and software tool that would estimate such models simultaneously both for monomers and their dimers have been missing.

**Results:**

We present an ADM-based mixture model for monomeric and dimeric TF-binding motifs and an expectation maximization algorithm MODER2 for learning such models from training data and seeds. The model is a mixture that includes monomers and dimers, built from the monomers, with a description of the dimeric structure (spacing, orientation). The technique is modular, meaning that the co-operative effect of dimerization is made explicit by evaluating the difference between expected and observed models. The model is validated using HT-SELEX and generated datasets, and by comparing to some earlier PPM and ADM techniques. The ADM models explain data slightly better than PPM models for 314 tested TFs (or their DNA-binding domains) from four families (bHLH, bZIP, ETS and Homeodomain), the ADM mixture models by MODER2 being the best on average.

**Availability and implementation:**

Software implementation is available from https://github.com/jttoivon/moder2.

**Supplementary information:**

Supplementary data are available at *Bioinformatics* online.

## 1 Introduction

Transcription factors (TFs) regulate the expression of their target genes by binding to specific DNA sequence segments (motifs) in the promoter and enhancer areas of the targets. Binding TFs may form clusters of two or more factors which makes the regulation combinatorial by nature (De Val *et al.*, 2008; Gordân and Siggers, 2013; Jolma *et al.*, 2015; Morgunova and Taipale, 2017; Panne *et al.*, 2007; Rodda *et al.*, 2005). Therefore it is of interest to develop models and learning algorithms of TF-DNA-binding motifs not only for monomeric binding but also for dimeric (and possibly higher order) co-operative binding of pairs of TFs. Such pairs can consist of two instances of the same factor (homodimer) or instances of two different factors (heterodimer). Models that represent dimeric motifs are composed of models for the monomeric motifs involved, plus a description of the structure of the dimer. Such a description represents the preferred relative spacings and orientations of the monomeric components of the dimer as well as models the co-operative effects.

Position-specific probability matrix (PPM) and the related position-specific weight matrix have been the standard model types for monomeric motifs (Stormo, 2000; Stormo *et al.*, 1986), and they have been used for modeling the dimers, too (Bi *et al.*, 2008; Bi and Rogan, 2004; Jankowski *et al.*, 2014; Kazemian *et al.*, 2013; Lu *et al.*, 2017; Whitington *et al.*, 2011). For example, a modeling procedure coMOTIF (Xu *et al.*, 2011) learns a comprehensive mixture model for motifs composed of two PPMs. Similarly, the method of Toivonen *et al.* (2018) learns a mixture composed in a modular fashion from one or more PPMs such that the structure of their preferred dimers is made explicit.

The standard PPM is an inhomogeneous Markov chain of order zero. PPM is a very simple model as it assumes that the bases in each individual position of the motif would contribute to the binding strength independently of each other. However, there can be dependencies between bases for various reasons [e.g. stacking interactions (Rohs *et al.*, 2010), amino acids may contact multiple bases simultaneously (Luscombe *et al.*, 2001), sequence-dependent multiple binding modes of a factor (Fordyce *et al.*, 2012; Meijsing *et al.*, 2009; Zuo and Stormo, 2014)]. Hence there has been a long-standing debate of whether PPMs suffice or should the motif model also represent dependencies between the bases (Benos *et al.*, 2002; Bulyk *et al.*, 2002; Man and Stormo, 2001; Zhao *et al.*, 2012). Markov models of order higher than zero are obvious candidates for more advanced models, capable of representing dependencies between two or more adjacent bases. There is accumulating evidence of better performance of higher order Markov models (Georgi and Schliep, 2006; Hannenhalli and Wang, 2005; Huang *et al.*, 2006; Maaskola and Rajewsky, 2014; Xing *et al.*, 2004; Zhao *et al.*, 2012). Very recently, Siebert and Söding (2016) give a robust expectation maximization (EM) algorithm (BaMM) for learning high-order Markov chains for monomeric motifs and demonstrate their superiority to order-zero models for several factors on ChIP-seq data. Models representing dependencies between any pair of positions, not only adjacent ones, have also been proposed, with evidence of superior performance in some cases (Barash *et al.*, 2003; Ben-Gal *et al.*, 2005; Omidi *et al.*, 2017; Santolini *et al.*, 2013; Sharon *et al.*, 2008; Siddharthan, 2010). On the other hand, the role of intra-motif dependencies might have been overestimated and the binding affinity interferences between multiple motifs should be given more emphasis (Eggeling, 2018).

This article presents a motif model for monomers and their dimers and the associated learning algorithm MODER2 that uses as its basic building blocks (inhomogeneous) first-order Markov chains. To the best of our knowledge, MODER2 is the first learning algorithm and software tool that uses first-order Markov modeling and discovers both monomeric and dimeric motifs. Matrices representing first-order Markov chains are called adjacent dinucleotide matrices (ADMs). Our motif model is a probabilistic mixture that includes one or more monomeric ADMs and all their dimers, with a description of dimer structure (spacing and orientation).

Modeling technique is modular in the sense that it uses an explicit representation of how each observed dimeric motif deviates from what is expected were the dimer motif just a ‘product’ of independent monomers, that is, the co-operative effects (multimotif interferences) of dimerization on binding affinities are discovered. This feature is consistent with recent observations in a number of dimeric cases of TF binding, that the specificity of the dimeric motif differs notably from what would be expected if the two factors would bind to DNA independently of each other (Isakova *et al.*, 2016; Jolma *et al.*, 2013, 2015).

MODER2 learns all components of the motif model in the same probabilistic framework, hence utilizing all training data symmetrically. Accurate learning of monomeric motifs is possible such that the noise from dimeric instances is minimized. This differs from the common way of learning motifs, in which one tries to discover only one motif at a time. Then, if the training data contains instances of dimeric motifs with the monomer as a half-site, the resulting model for a monomeric motif becomes an average of the instances from monomers and various dimers and hence can be inaccurate.

The MODER2 learning algorithm belongs to the EM algorithms, with additional techniques to improve the convergence, modularity and robustness of the search. Most important of these is the restriction of learning to a Hamming neighborhood of a seed (Toivonen *et al.*, 2017), which is here generalized for first-order Markov chains. Initiated by Lawrence and Reilly (1990), the EM algorithm has been extensively utilized for learning TF-binding motifs (Bailey *et al.*, 2009; Bailey and Elkan, 1995; Cardon and Stormo, 1992; Li, 2009; Mercier *et al.*, 2011; Quang and Xie, 2014; Reid and Wernisch, 2014; Xu *et al.*, 2011; Zhang *et al.*, 2013).

To validate MODER2, we report some motif discovery experiments using generated data as well as data from HT-SELEX. To demonstrate modular analyses possible with MODER2, we analyze TFs HNF4A and ARGFX. For HNF4A, we construct dimeric binding motifs of order one in three different ways and compare with the corresponding order-zero motifs. Then we compare the performance of MODER2 with MODER (order-zero models), BaMM [models of order one and two (Siebert and Söding, 2016)] and InMoDe [variable order models (Eggeling *et al.*, 2017)] on 314 HT-SELEX datasets for 233 TFs (or their DNA-binding domains) from four families (bHLH, bZIP, ETS and Homeodomain). The higher order models are observed to explain training data on the average better than the order-zero models.

While our validation tests use HT-SELEX data, MODER2 can be used on other training data such as ChIP-seq datasets as well. The training data should only be big enough to avoid over-fitting as the motif models learned can have quite a high number of parameters.

## 2 Motif model

Our model for TF-binding motifs is a probabilistic mixture composed of models for monomeric motifs and of models for dimeric motifs that are built from the monomeric models. We model monomeric motifs with inhomogeneous Markov chains of order one, represented as matrices we call ADMs. Each dimeric motif included in the mixture is represented as an ADM that is composed of a pair of monomeric ADMs, with associated information on the relative orientation and spacing of the two monomeric ADMs, and with the gap between the ADMs filled with the background model. The monomeric components of a dimer need not be spatially separate but their sites may overlap; such overlaps have been observed, for example in Jolma *et al.* (2015) and LaRonde-LeBlanc and Wolberger (2003).

If the two monomers of a dimer do not overlap and have a long gap in between (say, at least four as in our implementation), then the dimeric distribution is just the ‘product’ of the two monomer ADMs, that is, the model assumes that there is no co-operative interference affecting the independence of the two binding profiles. However, if the monomers overlap or the gap between them is short, then the binding profiles of the two monomers do not necessarily remain independent. The components of a dimer may interact because the components physically contact each other, or the interaction is DNA mediated (Jolma *et al.*, 2015). Therefore the model allows deviating from pure reduction to monomer ADMs. In this case it also represents, using the deviation component, how the ADM learned from data differs from the ‘product’ of monomer ADMs which would be the expected dimer model were there no interactions.

**Fig. 1.**
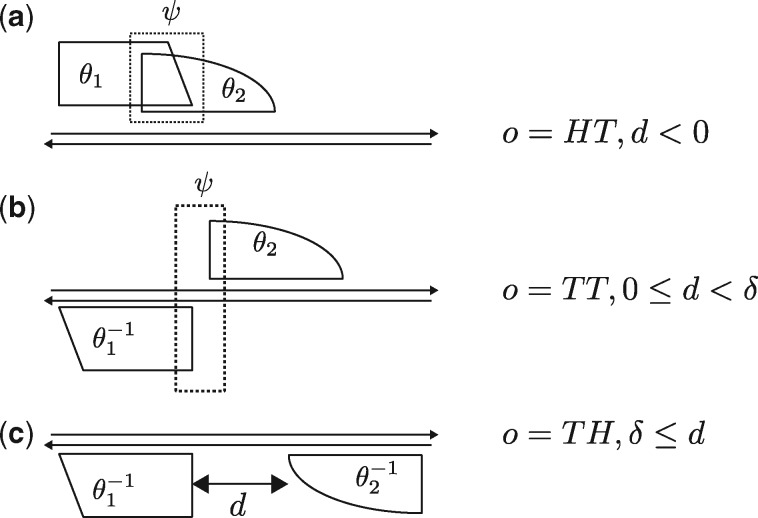
Parametrization of dimeric structures. Parameter *d* is the spacing (signed distance from the right end of the first monomer to the left end of the second monomer) of a dimer. Parameter δ is the lower bound such that monomers separated by a space d≥δ are assumed independent. Monomers are assumed dependent if d<δ. In this case, to represent monomer interferences, the model includes the bridging component ψ that covers the bridging segment, marked with the dotted box, of the dimer. (**a**) A dimer with overlapping monomers. Spacing *d* is negative and the length of the overlap is |d|. Orientation between monomer ADMs θ_1_ and θ_2_ is head-to-tail (HT). Shorthand notation of this dimer is 1,2,HT,d. (**b**) A non-overlapping but still close-by dimer in tail-to-tail (TT) orientation. Shorthand notation is 1,2,TT,d. The first monomer ADM $\theta_{1}^{-1}$ is the reverse complement of θ_1_. (**c**) A dimer in tail-to-head (TH) orientation. The first ADM $\theta_{1}^{-1}$ is the reverse complement of θ_1_, and the second ADM $\theta_{2}^{-1}$ is the reverse complement of θ_2_. The fourth possible orientation HH is not illustrated in the figure

More formally, our probabilistic mixture model is specified by parameters η=(θ,ψ,λ) where θ gives the monomeric ADMs and the background model used as the basic building blocks of the mixture, ψ gives the bridging component that models the bridging areas in the middle of dimers, and is used for discovering deviations from the expected model within dimers, and λ gives the mixture parameters that specify the relative strengths of the components of the mixture. Model η defines a probability distribution for sequences in the alphabet Σ of the model. We will use the DNA alphabet Σ={A, C, G, T} as default. Parametrization of the dimeric structures of the model has the alternatives HT, HT, TT, TH for the relative orientation *o* of the two components of a dimer (for homodimers only HT, HT, TT) as well as a parameter *d* giving the spacing between the components. Figure 1 gives an illustration of the parametrization.

The three parameter groups of η=(θ,ψ,λ) are as follows; see Supplementary Section S1 for full details.

Parameter $\theta =(\theta_{0},\ldots ,\theta_{p})$ gives the background distribution θ_0_ and *p* monomeric ADMs θ_*k*_. Background θ_0_ gives occurrence probabilities for alphabet symbols in sequence locations that are outside motif instances. Each ADM θ_*k*_, k≠0, is a $16\times \ell_{k}$ matrix

$${\text{θ}}_{k}={\left({\text{θ}}_{k}^{ab,h}\right)}_{a,b\in \Sigma ,\;h=1,\ldots ,\ell_{k},}\;$$

that represents an order-one Markov chain $\left(X_{1},\ldots ,X_{\ell_{k}}\right)$. The probability of a sequence $a=a_{1}a_{2}\cdots a_{\ell_{k}}$ given by ADM θ_*k*_ is

$$\begin{array}{cc} P(X=a) & =\prod_{1\leq h\leq \ell_{k}}P(X_{h}=a_{h}|X_{h-1}=a_{h-1})\\ & =\prod_{1\leq h\leq \ell_{k}}\theta^{a_{h-1}a_{h},h}. \end{array}$$

A dimeric ADM model composed of monomer ADMs $\theta_{k_{1}}$ and $\theta_{k_{2}}$, with orientation *o* and spacing *d*, is denoted as $\tau_{k_{1}k_{2}od}$. In the independent case *d* is ≥δ where threshold δ has default value 4. Then $\tau_{k_{1}k_{2}od}$ is composed of $\theta_{k_{1}}$ and $\theta_{k_{2}}$ in relative orientation *o* and with *d* columns of background in between. In the dependent case, *d* is less than δ and ψ gives the so-called bridging ADMs that model the segment of dimeric motifs in which we anticipate deviations from independence of monomer motifs. The |d|+2 columns in the middle of $\tau_{k_{1}k_{2}od}$ constitute the bridging ADM model, given by parameter $\psi_{k_{1}k_{2}od}$, and the rest of $\tau_{k_{1}k_{2}od}$ comes from the flanks of $\theta_{k_{1}}$ and $\theta_{k_{2}}$. The actual value of a deviation $\kappa_{k_{1}k_{2}od}$ is a derived parameter obtained as the difference of $\psi_{k_{1}k_{2}od}$ and the expected model.

Mixing parameters $\lambda =\{\lambda_{k}:k\in \text{monomers}\;\text{and}\;\text{dimers}\}$ give the probability of each component of the mixture. For each two monomer motifs, $\theta_{k_{1}}$ and $\theta_{k_{2}}$, the array $\left(\lambda_{k_{1}k_{2}od}\right)$ of mixing parameter values for different orientations *o* and spacings *d* of their dimers is called the co-operative binding table (COB table) of motifs $\theta_{k_{1}}$ and $\theta_{k_{2}}$. The values in a COB table indicate the orientation and spacing preferences of the dimeric structures that are composed of $\theta_{k_{1}}$ and $\theta_{k_{2}}$.

**Fig. 2.**
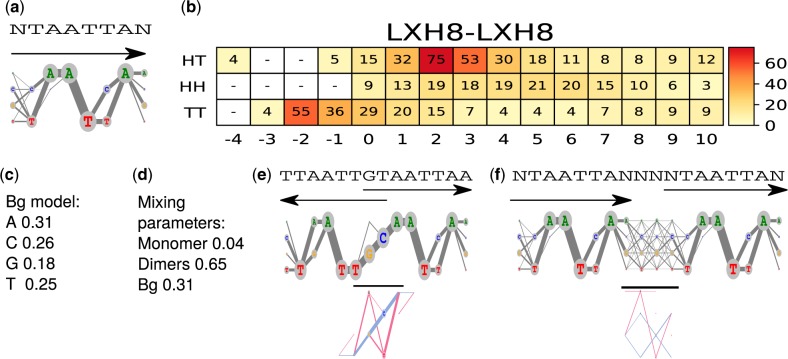
Mixture of ADMs for factor LHX8. The model was learned by MODER2 from a HT-SELEX dataset (ERR194392, 277 692 reads of length 40). ADMs are visualized as ‘river-lake logos’ (Morgunova *et al.*, 2015) in which nucleotides are shown within a circle whose radius is proportional to the probability of the nucleotide, and the edges connecting adjacent nucleotides have thickness proportional to the probability of the corresponding dinucleotide. (**a**) Monomeric ADM θ_1_ with original seed NTAATTAN. (**b**) Heat map of COB table $\left(\lambda_{1,1,o,d}\right)$ for homodimers of LHX8, giving the break-down into individual dimers and indicating that $\tau_{1,1,TT,-2}$ (panel e), $\tau_{1,1,HT,2}$ (panel f) and $\tau_{1,1,HT,3}$ are the strongest dimers. The values in the table are given in integer multiples of 0.001, horizontal axis gives the spacing *d*, and cells with no value indicate that the corresponding dimeric cases were pruned during the EM search; see Pruning the search subsection. (**c**) Background ADM θ_0_. (**d**) Mixture breakdown into one monomer, all dimers, and background. For example, 0.65 is the sum of dimeric mixing parameters λ_*k*_ that are in COB table b. (**e**) Dimeric ADM $\tau_{1,1,TT,-2}$ with (above) the combined seed and arrows indicating the orientation, and (below) horizontal bar indicating the bridging segment, and deviation $\kappa_{1,1,TT,-2}$ with positive values visualized in blue and negative values in red. For example, the right-most red edge for dinucleotide TA indicates that the quite high probability of the dinucleotide in the expected model vanishes in the observed model. (**f**) Dimeric ADM $\tau_{1,1,\text{HT},2}$ and its deviation

Figure 2 illustrates our model for binding motifs of TF LHX8. Note that we visualize both τ and κ.

This modeling framework can be varied by specifying explicitly the pairs $\theta_{k_{1}}$ and $\theta_{k_{2}}$ of monomer ADMs whose dimers are included in the mixture. In the *dimeric mode*, there is at least one such pair, in the *monomeric mode* there is no pair, that is the model is a mixture of monomeric ADMs only.

## 3 Materials and Methods

### 3.1 EM algorithm for learning the model

Given a set of training sequences that contain enriched motif instances, MODER2 (MOtif DEtectoR) learning algorithm finds the parameters (θ,ψ,λ) of all model components simultaneously, by optimizing the alignment of the training data with the model using maximum likelihood estimation. The ZOOPS (zero or one occurrence per sequence) model of alignment is used (Bailey and Elkan, 1995). The EM search is initialized with user-given seed sequences for the monomeric motifs to be learned, and the search is restricted to a user-given range of spacings and orientations of dimers.

A detailed description of the MODER2 algorithm is given in Supplementary Section S2.

### 3.2 Implementation

In this section, we give practical details of our software implementing the MODER2 algorithm and provide some modifications to improve its efficiency. The implementation includes both order-one (ADM) and order-zero (PPM) versions of the method.

**Input** MODER2 takes the following input data:

1. Training data *X* that consists of DNA sequences $X_{1},X_{2},\ldots ,X_{n}$, with lengths $|X_{i}|=L_{i}$ for i=1,…,n.
2. Seeds $s_{1},s_{2},\ldots ,s_{p}$. Each *s_k_* is an IUPAC sequence of length $|s_{k}|=\ell_{k}$. Seeds should be high-affinity representative sequences, one for each monomeric motif to be learned. They will be used for constructing initial values for ADMs θ_*k*_.
3. Set $R\subset {\left\{1,2,\ldots ,p\right\}}^{2}$ of pairs that gives the TF combinations to be learned. Only dimers of monomeric motifs $\theta_{k_{1}}$ and $\theta_{k_{2}}$ such that (*k*_1_, *k*_2_) is in *R* will be learned; for each (*k*_1_, *k*_2_) in *R*, parameters $d_{\text{min}}(k_{1},k_{2})$ and $d_{\text{max}}(k_{1},k_{2})$ give the interval of spacings of such dimers (default interval is $\left[-\min(\ell_{k_{1}},\ell_{k_{2}})/2,10\right]$); and parameter δ≥0 (default value 4) gives the minimum spacing such that the monomer motifs of a dimer are assumed independent if the gap of a dimer is ≥δ. If *R* is empty, then we have the monomeric mode and otherwise the dimeric mode of learning.
4. Maximum number of EM iterations, maxiter (default value 150) and the convergence threshold for parameter change in consecutive EM iterations, *ϵ* (default value 0.001).
5. Hamming radius ρ (default value 2 for PPMs and 3 for ADMs) used in seed-driven pruning of the EM search (see Pruning the search section).

**Output** MODER2 outputs the following results:

1. Monomer ADMs $\theta_{1},\ldots ,\theta_{p}$,
2. Monomer fractions $\lambda_{1},\ldots ,\lambda_{p}$,
3. Deviation matrices $\kappa_{k_{1},k_{2},o,d}$ for all $(k_{1},k_{2})\in R$, orientations *o*, and spacings d<δ,
4. The COB tables $\left(\lambda_{k_{1},k_{2}}\right)$ for all $(k_{1},k_{2})\in R$.

#### 3.2.1 Pruning the search

The implementation has some modifications to the pure EM framework in order to speed-up the search and to utilize prior knowledge of data quality as follows, more details given in Supplementary Section S3.

- At the start of the EM search, the mixture to be learned includes all dimers that are possible within the user-given range of gap lengths. Many of them are eventually not present in the training data. As soon as the mixing parameter of a dimer gets very small, such a weak dimer is removed from the mixture.
- Monomeric ADMs are not learned from the full data but only from monomeric occurrences of the monomers and from dimeric occurrences of the monomer such that the spacing between the components is large enough, the smallest such spacing given by input parameter δ. Such isolated occurrences within a dimer are supposed to give the best data for a monomer ADM, not distorted by close-by other sites such as the other component of a dimer. If the input parameter *R* is empty, that is no dimers are learned, then the full data are used for learning the monomeric ADMs.
- A TF may have two different binding motifs whose consensus sequences are only a few Hamming steps apart. To minimize disturbance from such similar motifs and from the background, MODER2 restricts the learning of ADMs to high-affinity training sequences. Such sequences are identified by the heuristic rule that they are in small Hamming neighborhood of the consensus sequences (sequences with highest probability) of the ADMs of the previous EM iteration (Toivonen *et al.*, 2017). The radius ρ of the Hamming neighborhood is a user-given parameter (default value 2 for PPMs and 3 for ADMs). Derivation and pseudocode of the method are given in Supplementary Section S3.

#### 3.2.2 Visualization and post-processing tools

The MODER2 algorithm is implemented in C++ and is available from GitHub. The package also contains tools to visualize the binding models and COB tables. Moreover, a post-processing tool is provided that selects from the model learned by MODER2 a submodel that consists of the strongest components of the mixture. Given a threshold (default 85%), the tool constructs a submodel by selecting the components of the original mixture in decreasing order of their mixing parameter λ until the fraction of the signal covered by the selected components reaches the threshold. The submodel is the final result of the motif learning procedure. With a suitable ADM scanning tool (e.g. Korhonen *et al.*, 2016), it can be applied for predicting putative motif instances elsewhere.

## 4 Results

### 4.1 Sanity checks with generated data

As an initial sanity test, we generated a dataset using a motif model, and checked that MODER2 is able to learn the model back from the generated data. We took monomeric ADM of HOXB13 (obtained earlier from SELEX data with MODER2 using seed CYMRTAAAA) and created homodimeric ADMs HT 4, HH 4, HH 2, TT 2 and HH 5 as the expected models (see Supplementary Section S4). To dimers HH 4 and HH 2, we further added deviation from the expected model by hand (see Supplementary Fig. S1). Three variants of this model were used, with different total signal fractions 0.03, 0.30 and 0.90. As an example, for total signal fraction 0.30 the model had the following component strengths: uniform background (λ=0.70), and ADMs for homodimers HT 4 (λ=0.061), HH 4 (0.055), HH 2 (0.068), TT 2 (0.034) and HH 5 (0.082); see Supplementary Figure S1, panel (i).

Using this model, 100 000 sequences of length 40 bp were generated. Given a seed CYMRTAAAA and Hamming radii ρ=2,3,…,9, and ∞, MODER2 accurately relearned the model from this data when total signal fraction was 0.3 or 0.9: the learned parameters differed from the original at most by 0.188 (for ρ* *= 3) in weighted maximum norm (Supplementary Section S1), and for larger radii, the difference was smaller, radii 7 and 8 giving the smallest differences; see Supplementary Table S2. For the low signal fraction 0.03, separating the signal from the background sometimes failed (ρ=5,7,∞).

Next we demonstrated that restriction to a small Hamming neighborhood may improve results. Data were generated using five models, each being a mixture of two ADMs. The ADMs were selected such that their consensus sequences are close to each other (Hamming distance at most 4 in most cases). Using each model, five datasets with respective monomer fractions 0.005, 0.015, 0.05, 0.15 and 0.45 were generated. From each dataset, MODER2 learned back the generating two monomer ADMs, using Hamming radii 2, 3, 4 and 5. The results, shown in Supplementary Figure S2, indicate that for low signal fraction, the restriction to a small Hamming neighborhood gives the most accurate results while for high signal fraction this effect disappears.

### 4.2 ADM versus PPM motifs of HNF4A and ARGFX

#### 4.2.1 HNF4A

Next we compare order-one and order-zero binding motifs of TF HNF4A and analyze what is the most economical representation of the underlying signal. HNF4A is known to bind as a dimer, predominantly as direct repeats with spacing of one nucleotide (or rarely two) (Badis *et al.*, 2009; Ellrott *et al.*, 2002; Jiang and Sladek, 1997). ADM models for HNF4A motifs were learned by MODER2 from HT-SELEX data ERX169045 [Jolma *et al.* (2013), 655 432 reads of length 40]. To eliminate boundary effects due to the barcode and primer sequences flanking the random window of 40 bases, we included 6 and 3 bases long constant flanks in the beginning and end of the reads, respectively, making them 49 bases long.

We made three different analyzes with the following seeds.

(E1) Two monomers and their dimers. Seeds RGKYCA and AGTCCA for the monomers, that is possibly different monomers represent the left and right half-sites of the dimer. This is similar to the PPM analysis of HNF4A in (Toivonen *et al.*, 2018).

(E2) One monomer and its dimers. Seed RGKYCA for the monomer, that is the same monomer represents both half-sites.

(E3) One long monomer without dimers. Seed RGKYCANRGKYCA of length 13, that is the only monomer is intended to represent the strongest dimer with half-sites of length 6 and a gap of one nucleotide in between.

**Fig. 3.**
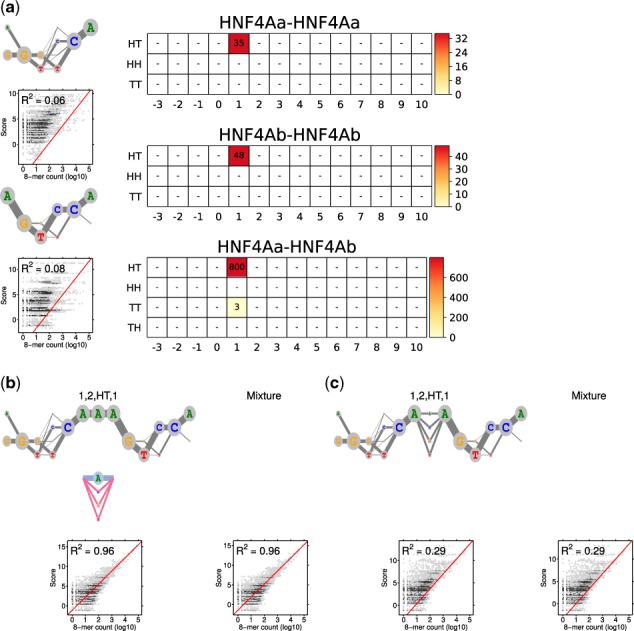
Modularity analysis of HNF4A using two monomers, ADM case. (**a**) Monomer ADMs θ_1_ and θ_2_ ($\lambda_{1}=0.020,\lambda_{2}=0.002$) and the COB tables in units of integer multiples of 0.001, as learned from data by MODER2. Since all the mixing parameters are in the same scale, comparison of λ values is possible between COB tables. Also shown is *R*^2^ correlation analysis for the two monomer models. (**b**) The 85% rule gives final mixture that includes the dimeric ADM $\tau_{1,2,\text{HT},1}$ only. Its deviation is depicted below the ADM logo. The mixture has the same correlation as its only component. (**c**) Correlation analysis as in (b) but for the ADM $E_{1,2,\text{HT},1}$ that is expected under the independence assumption. The *R*^2^ values for the learned and expected ADM differ remarkably, reflecting the large deviation between the learned and the expected ADMs. The expected model does not detect the AAA sequence connecting the half-sites

Figure 3 illustrates the ADM model resulting from case (E1). Supplementary Figure S3 repeats the PPM model from earlier experiment (Toivonen *et al.*, 2018) for comparison. Results for (E2) and (E3) are shown in Supplementary Figures S4 and S5. The quality of models is measured and illustrated in scatter plots using correlation (*R*^2^) between occurrence counts and model scores of 8-mers of HT-SELEX data, as explained in Supplementary Section S5.

Experiment (E1) produces a model with largest number of parameters and also the highest correlation $R^{2}=0.96$. The same correlation is achieved by (E3), and third is (E2) with $R^{2}=0.83$. Hence the only monomer of (E3), that in effect represents the strongest dimer found by (E1) and (E2), reaches alone an *R*^2^ that is as good as or better than the *R*^2^ of the richer mixture models of (E1) and (E2).

When comparing the PPM model (Supplementary Fig. S3) with the ADM models, one observes that the only ADM of (E3) presents quite accurately the three dimeric PPMs of the PPM model, because the three strongest paths through that ADM give the dominant sequences of the PPMs.

#### 4.2.2 ARGFX

**Fig. 4.**
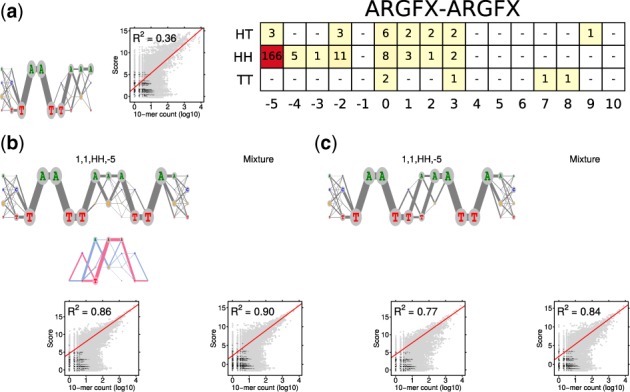
Modularity analysis of ARGFX, ADM case. (**a**) Monomer ADM θ_1_ (λ=0.188), the COB table in the units of integer multiples of 0.001, and *R*^2^ correlation analysis. (**b**) Dimeric ADM $\tau_{1,1,HH,-5}$, its deviation and *R*^2^ correlation analysis. The 85% rule gives mixture that includes the monomeric ADM of (a) and the dimeric ADM of (b) (λ=0.166). The *R*^2^ analysis of the mixture. (**c**) Correlation analysis as in (b) but for the ADM $E_{1,1,HH,-5}$ that is expected under the independence assumption. Correlation analysis of the mixture as in (b) but with $E_{1,1,HH,-5}$ instead of $\tau_{1,1,HH,-5}$

ADM and PPM models for TF ARGFX were learned from HT-SELEX data ERX1081111 [Yin *et al.* (2017), 131 066 reads of length 40]. Figure 4 illustrates the ADM model and Supplementary Figure S6 the PPM model. Full mixture model is the most accurate model in both bases, with clear difference to individual components of the mixture and to the expected model. ADM and PMM models have the same consensus sequences, ADM being more accurate.

### 4.3 Performance comparison of MODER2, BaMM and InMoDe

Here, we compare the accuracy of motif models learned by MODER2 (models of order one and zero, Toivonen *et al.*, 2018), BaMM (models of order one and two) and InMoDe (variable order models of order at most 2). BaMM is a recent higher order Markov learning algorithm that compares favorably with several earlier ones (Siebert and Söding, 2016). InMoDe learns inhomogeneous parsimonious Markov models with varying context lengths (Eggeling *et al.*, 2017).

We took the HT-SELEX datasets published with associated seeds in Yin *et al.* (2017) from which we selected all datasets in the bHLH, bZIP, ETS and Homeodomain families. These families were selected as by the analysis of Jolma *et al.* (2013) these are the largest monomer-rich and dimer-rich families, large families giving enough data for finding possible differences between model accuracies within each family. We ignored the large ZnF family as its HT-SELEX success rate was low, meaning that the available data would not represent the family well. Moreover, if the seed of a dataset was an obvious dimeric seed (with IUPAC Ns in the middle), we split it into two seeds for half-sites as our tool is for finding dimers that are composed of monomers. Finally, if a dataset had several associated seeds, we only used the shortest one with highest count in the data. After this, we had 314 datasets (number of reads in a dataset between 95 485 and 1 294 346, read length always 40 bp), of which 67, 49, 33 and 165 datasets belong to bHLH, bZIP, ETS and Homeodomain families, respectively. Details of the 314 datasets are given in Supplemental File Data-and-Experiments.

For these datasets, order-one and order-zero models were learned by MODER2, both in monomeric mode (mixture of monomers) and in dimeric mode (mixture of monomers and their dimers), order-one and order-two models were learned by BaMM, and variable order models were learned by InMoDe. The quality of all models was measured using correlation (*R*^2^) between occurrence counts and model scores of 10-mers of training data (see Supplementary Section S5).

**Fig. 5.**
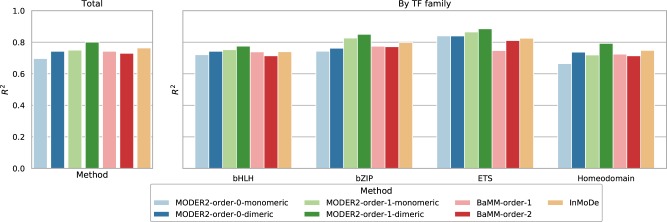
Median correlations (*R*^2^) for the tested methods in total and in four families. For more details, see Supplementary Table S4

Figure 5 gives the median *R*^2^ values for the seven models learned, in the 314 datasets and in each of the four TF families. Supplementary Table S4 gives numeric values (and average *R*^2^ values). Supplementary File Data-and-Experiments gives *R*^2^ values for each model and each dataset. Supplementary File All-Models gives matrices representing 314 dimeric models of order 1 learned by MODER2.

In Figure 5, the performance differences between the tested methods are quite small in general. The order-one dimeric models by MODER2 have consistently the highest median *R*^2^, InMoDe being the next, and the order-zero monomeric models by MODER2 (i.e. classic PPMs) having expectedly the lowest *R*^2^, sometimes (e.g. in Homeodomain) quite clearly.

It should be emphasized that the modeling techniques compared above are qualitatively different: MODER2 discovers in dimeric mode mixture models (mixtures of monomers and their dimeric combinations) that represent more complex motif structures than the one-motif models produced by BaMM and InMoDe. Therefore the above comparison that uses the same *R*^2^ framework for all models can only indicate how well different models fit the training data but it ignores essential qualitative differences.

## 5 Discussion

We presented a modeling framework and an EM learning algorithm MODER2 for *de novo* detection of TF-binding motifs, represented as inhomogeneous order-one Markov chains. Our motif model is a probabilistic mixture of one or more monomeric motifs and their combinations (dimers), all learned simultaneously. Markov chains of order one have increasing evidence of outperforming the classic PPMs (Markov chains of order zero). Here they are used for the first time in full-fledged combinatorial modeling of motifs which uses monomeric motifs as basic modules and builds dimeric models from them.

Software implementation of the method (written in C++ on Linux platform and available in GitHub) is reasonably fast and can process quite large datasets. For example, it took 1 h 25 min 9 s wall-clock time and 11 h 00 min 47 s CPU time when run in parallel on eight cores to learn the model for HNF4A in Figure 3 from a 13 854 211 bp long HT-SELEX dataset. Seeds for initialization of the EM search can be taken from existing PPM motifs in databases or they can be extracted from the training data with seed-finding tools. One could simply take as the seed the most frequent ℓ-mer of the data where ℓ is the anticipated length of the motif.

Our modeling technique was validated using generated and HT-SELEX data for model training. Versatility of the technique was demonstrated by comparing order-one and order-zero motifs of TF HNF4A and ARGFX. We also compared order-one and higher-order motif models of four families of TFs and found that the order-one models are on average more accurate than order-zero models while models of order higher than one seem not to give much improvement.

## Funding

This work was supported by European Commission Framework Program 7 project SYSCOL [UE7-SYSCOL-258236]; Leverhulme Trust [VP1-2014-044 to E.U.]; Academy of Finland, ‘Finnish CoE in Tumor Genetics Research’ [312041]. 

*Conflict of Interest*: none declared. 

## Supplementary Material

btaa045_Supplementary_Data
