# Supplementary material for: MODER2: first-order Markov modeling and discovery of monomeric and dimeric binding motifs
Source: Bioinformatics. 2020 Jan 30;36(9):2690–6. doi: 10.1093/bioinformatics/btaa045 (PMC7203737; doi:10.1093/bioinformatics/btaa045)
Supplement: btaa045_Supplementary_Data [file btaa045_supplementary_data.zip › btaa045-Suppl_Data/moder2-supplement.pdf]

MODER2: First-order Markov Modeling and Discovery of Monomeric and Dimeric Binding Motifs

Supplementary Material
20.12.2019

Contents

S1 Structure of the motif model S1
S2 EM learning of the model S10
S3 Details of implementation S15
S4 Sanity checks with generated data S22
S5 Order-zero vs order-one motifs of HNF4A and ARGFX S25
S6 Performance comparison of MODER2, BaMM, and InMoDe S29
S7 Symbol definitions S30
References S32

S1 Structure of the motif model

The binding affinity model learned by MODER2, specified by parameters $\eta = (\theta, \psi, \lambda)$, gives a probability distribution for sequences in some alphabet Σ. We will always use the DNA alphabet $\Sigma = \{A, C, G, T\}$, but the model works for arbitrary alphabets.

Model η is a mixture of distributions for *monomeric sequences* that contain one occurrence of a monomeric motif, and distributions for *dimeric sequences* that contain two monomeric motifs in a specific relative orientation and spacing, and a distribution for *background sequences*. Monomeric distributions are built from the monomer models and the background. For all orientation and spacing alternatives between the two monomers in a dimer, dimeric distributions are built either from the monomer models and the background or from the model of the entire dimer and the background. Inhomogeneous first-order Markov chains are used as the monomer models. We call their matrix representation *Adjacent Dinucleotide Matrices* or ADMs for short. Inhomogeneous zero-order Markov models, i.e., the traditional

PPMs (Position-specific Probability Matrices) could be used as well. For completeness, our description below gives both alternatives.

The three parameter groups of $\eta = (\theta, \psi, \lambda)$ and the parametrization of the dimeric structures are defined in detail in the following subsections.

Monomeric models θ_k and background θ_0

Parameter $\theta = (\theta_0, \theta_1, \ldots, \theta_p)$ gives the background distribution θ_0 and p monomeric motifs θ_k.

For background we use mononucleotide model $\theta_0 = [\theta_0^{\mathrm{A}}, \theta_0^{\mathrm{C}}, \theta_0^{\mathrm{G}}, \theta_0^{\mathrm{T}}]^T$ which gives the occurrence probabilities of each alphabet symbol in any position that is outside the occurrences of monomers or dimers. The background model is position-independent.

Each monomeric model θ_k, $k \neq 0$, is represented by $H \times \ell_k$ matrix, where H is 4 for PPM type of models and 16 for ADMs. A PPM

$$\theta_k = \begin{bmatrix} \theta_k^{\mathrm{A},1} & \theta_k^{\mathrm{A},2} & \cdots & \theta_k^{\mathrm{A},\ell_k} \\ \theta_k^{\mathrm{C},1} & \theta_k^{\mathrm{C},2} & \cdots & \theta_k^{\mathrm{C},\ell_k} \\ \theta_k^{\mathrm{G},1} & \theta_k^{\mathrm{G},2} & \cdots & \theta_k^{\mathrm{G},\ell_k} \\ \theta_k^{\mathrm{T},1} & \theta_k^{\mathrm{T},2} & \cdots & \theta_k^{\mathrm{T},\ell_k} \end{bmatrix},$$

defines an inhomogeneous zero-order Markov chain $(X_1, \ldots, X_{\ell_k})$ such that $P(X_h = a) = \theta_k^{a,h}$ gives the probability for an alphabet symbol (nucleotide) a to occur in position h of the chain. The chain has independent variables, hence the probability of a sequence $x_1 x_2 \cdots x_{\ell_k}$ of ℓ_k alphabet symbols is

$$P(X_1 = x_1, \ldots, X_{\ell_k} = x_{\ell_k}) = \prod_{1 \leq h \leq \ell_k} P(X_h = x_h) = \prod_{1 \leq h \leq \ell_k} \theta_k^{x_h,h}.$$

The reverse complement θ_k^{-1} of θ_k is a PPM such that $\theta_k^{-1}[a,h] = \theta_k[\bar{a}, \ell_k - h + 1]$ for each a and h, where $\bar{a}$ is the complementary base of a (e.g., $\overline{\mathrm{A}} = \mathrm{T}$) and we have denoted $\theta_k^{a,h}$ by $\theta_k[a,h]$.

An ADM is matrix $\theta_k = [\theta_k^{ab,h}]$, where $1 \leq h \leq \ell_k$, and $a, b \in \Sigma$. It defines an inhomogeneous first-order Markov chain $(X_1, \ldots, X_\ell)$ such that $P(X_h = b | X_{h-1} = a) = \theta_k^{ab,h}$ gives the probability for an alphabet symbol b to occur in position h of the chain on the condition that the previous symbol was a. It is required that $\sum_b \theta_k[ab,h] = 1$ or that $\theta_k[ab,h] = 0$ for all b (which means that symbol a can not occur in position $h-1$). For convenience, the initial probability $P(X_1 = b \mid \cdot) = \theta_k^{\cdot b,1}$ of symbol b is repeated four times in the representation θ_k.

The probability of a sequence $x_1 x_2 \cdots x_{\ell_k}$ of ℓ_k alphabet symbols is

$$P(X_1 = x_1, \ldots, X_{\ell_k} = x_{\ell_k}) = \prod_{1 \le h \le \ell_k} P(X_h = x_h | X_{h-1} = x_{h-1}) = \prod_{1 \le h \le \ell_k} \theta^{x_{h-1} x_h, h}.$$

The reverse complement θ_k^{-1} of θ_k is an ADM such that

$$\theta_k^{-1}[ab, h] = \frac{\theta_k[\overline{ba}, \ell_k - h + 2] \theta_k[\bar{b}, \ell_k - h + 1]}{\theta_k[\bar{a}, \ell_k - h + 2]},$$

where $\overline{ab}$ denotes the complementary dinucleotide $\bar{a}\bar{b}$ (e.g., $\overline{\mathrm{AC}} = TG$). If the denominator is zero, we define $\theta_k^{-1}[ab, h] = 0$ for all $b \in \Sigma$. With the ADM models we have used above the notation $\theta^{a,h}$ for the probability of symbol a in position h, that is,

$$\theta^{a,h} = \theta[a, h] = \sum_{a_1, \ldots, a_{h-1} \in \Sigma} \theta[\cdot a_1, 1] \theta[a_1 a_2, 2] \cdots \theta[a_{h-1} a, h].$$

Weighted max norm

For PPMs θ_1 and θ_2 we define the *max norm distance* as

$$d(\theta_1, \theta_2) = \max_{a \in \Sigma, 1 \le h \le \ell} |\theta_1^{a,h} - \theta_2^{a,h}|,$$

and for ADMs θ_1 and θ_2 as a distance between dinucleotide probabilities:

$$d(\theta_1, \theta_2) = \max_{a,b \in \Sigma, 1 \le h \le \ell} |\theta_1^{a,h-1} \theta_1^{ab,h} - \theta_2^{a,h-1} \theta_2^{ab,h}|.$$

We sometimes call the above distances as the *weighted max norm distances*.

Dimer specification $k_1 k_2 od$

The model uses monomeric motifs θ_k as building blocks of dimeric motifs. The possible dimeric motifs are indexed with quadruples (k_1, k_2, o, d) which we abbreviate as $k_1 k_2 od$ (this should not be confused with the multiplication of these symbols). A dimer with index $k_1 k_2 od$ is composed of monomers θ_{k_1} and θ_{k_2} whose orientation is o and distance (spacing) from the end of θ_{k_1} to the start of θ_{k_2} is d, where $o = (o_1, o_2) \in \Omega_{k_1 k_2}$ and $d \in \Delta_{k_1 k_2}$. Because of co-operative binding effects, monomer motifs alone are not enough for building dimeric models. To model such effects we will use an additional model (see the next subsection) that covers the middle area of the dimer, called the *bridging segment*. Fig. 1 in the main text illustrates our parametrization of dimeric structures; c.f. (Bi and Rogan, 2004).

The set of possible pairwise orientations o is $\Omega_{k_1k_2} = \{\mathrm{HT}, \mathrm{HH}, \mathrm{TT}\}$ if $k_1 = k_2$ (*homodimer*), and $\Omega_{k_1k_2} = \{\mathrm{HT}, \mathrm{HH}, \mathrm{TT}, \mathrm{TH}\}$ otherwise (*heterodimer*). Table S1 describes different orientations $o = (o_1, o_2)$ giving the directions of motifs θ_{k_1} and θ_{k_2}. Note that for homodimers the orientations HT and TH are identical, and one can use HT to represent them both. We assume that motif θ_{k_1} always occurs before motif θ_{k_2} when moving from $5'$ end to $3'$ end and using motif start position as reference point. The reverse order of the two motifs transforms back to this case by considering the complementary strand. If the alphabet does not have the concept of *complement symbol*, like nucleotides have, then the set of possible orientations consists only of HT and TH.

The possible distances between the two occurrences are given as an interval $\Delta_{k_1k_2} = [\mathrm{dmin}(k_1,k_2), \mathrm{dmax}(k_1,k_2)]$. If $d \in \Delta_{k_1k_2}$ is non-negative, it gives the number of gap positions between the two occurrences. If $d < 0$, then the occurrences overlap by $|d|$ positions. The smallest possible distance $\mathrm{dmin}(k_1,k_2)$ has to be $> -\ell_{k_1}$. MODER2 implementation uses (optionally adjustable) default value $\mathrm{dmin}(k_1,k_2) = -\min(\ell_{k_1}, \ell_{k_2})/2$, that is, overlaps only up to half of the length of the monomers are allowed. The longest distance possible for sequences of maximum length $L_{\max}$ is $\mathrm{dmax}(k_1,k_2) = L_{\max} - \ell_{k_1} - \ell_{k_2}$.

We use parameter $\delta \geq 0$ to give the minimum spacing such that if the space between the two monomers of a dimer is $\geq \delta$ then the monomer profiles are assumed independent, i.e., in this case the model ignores the possible co-operative interactions that would change the binding preferences of the two TFs or the gap between them. Parameter δ is a user-given constant (default value $\delta = 4$).

In what follows, we refer to the available monomeric and dimeric motifs with index k that may belong to the following three separate sets:

$M = \{1, \ldots, p\}$: indices for monomeric motifs.

$D^+ = \{k_1k_2od : d \geq \delta, k_1, k_2 \in M\}$: indices for dimeric motifs whose monomers θ_{k_1} and θ_{k_2} have a gap of length $\geq \delta$ in between. This is called the *independent case*.

$D^- = \{k_1k_2od : d < \delta, k_1, k_2 \in M\}$: indices for dimeric motifs whose monomers θ_{k_1} and θ_{k_2} have a gap of length $< \delta$ in between. This is called the *dependent case*. Note that this case includes dimers whose monomers overlap.

Dimeric models $\tau_{k_1k_2od}$, bridging models $\psi_{k_1k_2od}$ and deviation matrices $\kappa_{k_1k_2od}$

We use $\tau_{k_1k_2od}$ to denote the model (which is a $H \times (\ell_{k_1} + \ell_{k_2} + d)$ matrix) for motif $k_1k_2od \in D^+ \cup D^-$. Each $\tau_{k_1k_2od}$ is a derived parameter, composed of free parameters, such that if $k_1k_2od \in D^+$ then $\tau_{k_1k_2od}$ is built from θ_{k_1}, θ_{k_2}, and background θ_0, and if $k_1k_2od \in D^-$ then $\tau_{k_1k_2od}$ is built from θ_{k_1}, θ_{k_2}, and the bridging PPM $\psi_{k_1k_2od}$ to be defined below. Constructions of $\tau_{k_1k_2od}$, in which we use shorthand notations $\theta_\alpha := \theta_{k_1}^{o_1}$ and $\theta_\beta := \theta_{k_2}^{o_2}$, are as follows.

Table S1: **Relative orientation of two motif occurrences within a dimer.**

Orientation o	Short-hand		o_1	o_2
Head-to-Tail	HT	$\rightarrow\rightarrow$	$+1$	$+1$
Head-to-Head	HH	$\rightarrow\leftarrow$	$+1$	-1
Tail-to-Tail	TT	$\leftarrow\rightarrow$	-1	$+1$
Tail-to-Head	TH	$\leftarrow\leftarrow$	-1	-1

Exponents o_1 and o_2 give the orientations of the first and the second model: for a model θ_k, θ_k^{+1} leaves the matrix intact but θ_k^{-1} takes the reverse complement.

Independent case: if $k_1k_2od \in D^+$, then we put simply

$$\tau_{k_1k_2od} = \theta_\alpha \bullet \theta_0' \bullet \ldots \bullet \theta_0' \bullet \theta_\beta \tag{S1}$$

where $\bullet$ concatenates matrices. The $H \times 1$ column-matrix θ_0' is θ_0 in the case of PPM, and the vertical concatenation of four copies of θ_0 in the case of ADM θ_0'. There are d column-matrices θ_0 in the middle of $\tau_{k_1k_2od}$, that is, the middle gap is filled with the background.

Dependent case: if $k_1k_2od \in D^-$, then a middle segment of $\tau_{k_1k_2od}$ is a free parameter learned from data: for $d < 0$, the columns that are on the overlap area (plus one more column on both sides) are free parameters, and for $0 \leq d < \delta$, the columns that are on the area between the monomers (plus one more column on both sides) are free parameters. This area of length $|d|+2$ in the middle of a dimer is called the *bridging segment*, and the $H \times (|d|+2)$ model for the bridging segment is called the *bridging model*. We let $\psi_{k_1k_2od}$ denote the bridging model. Now, the columns of $\tau_{k_1k_2od}$ that cover the bridging segment come from $\psi_{k_1k_2od}$ while the columns outside this segment are supposed to reduce to the monomer motifs, i.e., they are as in the implied prefix and suffix segments of monomer matrices $\theta_{k_1}^{o_1}$ and $\theta_{k_2}^{o_2}$. So we get

$$\begin{aligned} \tau_{k_1k_2od} = \theta_\alpha[\cdot, 1 : \ell_{k_1} + \min(d,0) - 1)] \bullet \psi_{k_1k_2od} \\ \bullet\, \theta_\beta[\cdot, \max(0,-d) + 2 : \ell_{k_2}]. \end{aligned} \tag{S2}$$

Next we make it explicit how $\tau_{k_1k_2od}$ differs from the "product" model, which is the expected model were the monomer motifs mutually independent in the dimer. We denote such an *expected* model as $E_{k_1k_2od}$. It models sequences $X_1X_2\cdots X_{\ell_{k_1}+\ell_{k_2}+d}$ such that monomeric motifs θ_α and θ_β have independent instances at distance d from each other, with an occurrence of θ_α at the left end and θ_β at the right end of the sequence.

Expected model for non-overlapping case. Let $0 \leq d < \delta$. Consider the occurrence prob-

ability $P(X_h = b|X_{h-1} = a)$ of symbol b in position h of the expected model. Obviously, if $h \leq \ell_{k_1}$, then

$$P(X_h = b|X_{h-1} = a) = \begin{cases} \theta_\alpha[b,h] & \text{for PPM} \\ \theta_\alpha[ab,h] & \text{for ADM;} \end{cases} \tag{S3}$$

if $\ell_{k_1} < h \leq \ell_{k_1} + d$, then $P(X_h = b|X_{h-1} = a) = \theta_U[b]$, i.e., we expect to see the uniform background ($\theta_U[b] = 1/4, b \in \Sigma$) distribution between the two motifs; and if $h > \ell_{k_1} + d$, then

$$P(X_h = b|X_{h-1} = a) = \begin{cases} \theta_\beta[b,h-\ell_{k_1}-d] & \text{for PPM} \\ \theta_\beta[ab,h-\ell_{k_1}-d] & \text{for ADM;} \end{cases} \tag{S4}$$

This means that $E_{k_1k_2od}$ is just θ_α followed by d columns, each equal to θ_0', followed by θ_β; c.f., the definition of $\tau_{k_1k_2od}$ in the independent case (S1).

Expected model for overlapping PPMs. Let $d < 0$, i.e., the monomeric PPMs overlap by $|d|$ symbols. Consider again the probability $P(X_h = b)$. If $h \leq \ell_{k_i} + d$, then

$$P(X_h = b) = \theta_\alpha[b,h], \tag{S5}$$

and hence the hth column of the expected PPM is $E_{k_1k_2od}[\cdot,h] = \theta_\alpha[\cdot,h]$. Similarly, if $h > \ell_{k_1}$, then

$$P(X_h = b) = \theta_\beta[b,h-(\ell_{k_1}+d)], \tag{S6}$$

and hence $E_{k_1k_2od}[\cdot,h] = \theta_\beta[\cdot,h-(\ell_{k_1}+d)]$.

In the remaining case we have $\ell_{k_1} + d < h \leq \ell_{k_1}$, and the hth symbol b belongs to the area where the two motifs overlap. Hence b is generated by both θ_α and θ_β, under the condition that both generate the same symbol because in the overlapping area the two motifs have to coincide. Therefore we let $P(X_h = b)$ equal $\theta_\alpha[b,h]\theta_\beta[b,h-(\ell_{k_1}+d)]$, normalized by the condition that both motifs generate the same symbol. This gives

$$P(X_h = b) = \frac{\theta_\alpha[b,h]\theta_\beta[b,h-(\ell_{k_1}+d)]}{\sum_{c\in\Sigma}\theta_\alpha[c,h]\theta_\beta[c,h-(\ell_{k_1}+d)]}, \tag{S7}$$

and therefore the hth column becomes

$$E_{k_1k_2od}[\cdot,h] = \frac{\theta_\alpha[\cdot,h] \times \theta_\beta[\cdot,h-(\ell_{k_1}+d)]}{\sum_{c\in\Sigma}\theta_\alpha[c,h]\theta_\beta[c,h-(\ell_{k_1}+d)]}, \tag{S8}$$

where $\times$ denotes element-wise product.

Expected model for overlapping ADMs. In the ADM case the dependencies between adjacent positions complicate the derivation of $E_{k_1k_2od}$ somewhat. Let $d < 0$, i.e., the monomeric ADMs overlap by $|d|$ symbols. It is convenient to make θ_α and θ_β to overlap throughout. This is done by padding them, respectively, by $\ell_\beta + d$ and $\ell_\alpha + d$ columns of uniform background $\theta_U[ab] = 1/4, a, b \in \Sigma$ to get equally long matrices $\Theta_\alpha = \theta_\alpha \theta_U^{\ell_\beta+d}$ and $\Theta_\beta = \theta_U^{\ell_\alpha+d}\theta_\beta$. When aligned, these matrices retain the original overlap of length $|d|$ and outside it the padded flanks have neutral effect on the joint model we want to construct. We get $E_{k_1k_2od}$ by forcing these two ADMs to produce the same sequences.

We use general formulation of the forcing technique. Let $X = (X_1, \ldots, X_\ell)$ be a Markov chain with state space Σ, initial probabilities $P(X_1 = a)$, and transition probabilities $P(X_{h+1} = b|X_h = a)$ for all $h \in \{1, \ldots, \ell - 1\}$ and $a, b \in \Sigma$. Define similarly Markov chain $Y = (Y_1, \ldots, Y_\ell)$. The product Markov chain $W = (W_1, \ldots, W_\ell)$ is defined over state space Σ^2 as follows:

$$P(W_1 = (a, a')) = P(X_1 = a)P(Y_1 = a')$$

and

$$P(W_{h+1} = (b, b')|W_h = (a, a')) = P(X_{h+1} = b|X_h = a)P(Y_{h+1} = b'|Y_h = a').$$

We use the shorthand notation $W_h = a_h$ to mean $W_h = (a_h, a_h)$.

We define random vector $Z = (Z_1, \ldots, Z_\ell)$ as an "intersection-product" of X and Y by

$$P(Z = (a_1, \ldots, a_\ell)) := P(W = (a_1, \ldots, a_\ell)|X = Y), \tag{S9}$$

where $a_h \in \Sigma$ for all $1 \leq h \leq \ell$, and the shorthand notation $W = (a_1, \ldots, a_\ell)$ means $W_h = a_h$ for all $1 \leq h \leq \ell$. The chain rule gives $P(Z = (a_1, \ldots, a_\ell)) = P(Z_1 = a_1)P(Z_2 = a_2|Z_1 = a_1) \cdots P(Z_\ell = a_\ell|Z_1 = a_1, \ldots, Z_{\ell-1} = a_{\ell-1})$. Next we show that Z is in fact a Markov chain (of order one). First we will define a helper function

$$\begin{aligned}
f(a_h, h) &:= P(X_{h+1} = Y_{h+1}, \ldots, X_\ell = Y_\ell | W_h = a_h) \\
&= \sum_{b_{h+1}, \ldots, b_\ell \in \Sigma} P(W_{h+1} = b_{h+1}, \ldots, W_\ell = b_\ell | W_h = a_h), \\
&= \sum_{b_{h+1}, \ldots, b_\ell \in \Sigma} P(W_{h+1} = b_{h+1} | W_h = a_h) P(W_{h+2} = b_{h+2}, \ldots, W_\ell = b_\ell | W_{h+1} = b_{h+1}), \\
&= \sum_{b_{h+1} \in \Sigma} P(W_{h+1} = b_{h+1} | W_h = a_h) \sum_{b_{h+2}, \ldots, b_\ell \in \Sigma} P(W_{h+2} = b_{h+2}, \ldots, W_\ell = b_\ell | W_{h+1} = b_{h+1}) \\
&= \sum_{b_{h+1} \in \Sigma} P(W_{h+1} = b_{h+1} | W_h = a_h) f(b_{h+1}, h+1),
\end{aligned}$$

for all $a_h \in \Sigma, 0 \leq h \leq \ell$. Note that $f(a_\ell, \ell) = P(X_{\ell+1} = Y_{\ell+1} = \varepsilon | W_\ell = a_\ell) = 1$ and $f(a_0, 0) = P(X = Y)$. So, we can compute the values of array f starting from $h = \ell$ towards $h = 0$.

Proposition 1. *Z is a Markov chain of first order with transition probabilities*

$$P(Z_h = a_h | Z_{h-1} = a_{h-1}) = P(W_h = a_h | W_{h-1} = a_{h-1}) \frac{f(a_h, h)}{f(a_{h-1}, h-1)}.$$

Proof. We show that

$$P(Z_h = a_h | Z_1 = a_1, \ldots, Z_{h-1} = a_{h-1}) = P(Z_h = a_h | Z_{h-1} = a_{h-1})$$

for all $h \in \{2, \ldots, \ell\}$. Direct computation gives

$$\begin{aligned}
P(Z_h = a_h | Z_1 = a_1, \ldots, Z_{h-1} = a_{h-1}) &= \frac{P(Z_1 \cdots Z_h = a_1 \cdots a_h)}{P(Z_1 \cdots Z_{h-1} = a_1 \cdots a_{h-1})} \\
&= \frac{\sum_{b_{h+1},\ldots,b_\ell \in \Sigma} P(Z_1 \cdots Z_i Z_{h+1} \cdots Z_\ell = a_1 \cdots a_i b_{h+1} \cdots b_\ell)}{\sum_{b_h,\ldots,b_\ell \in \Sigma} P(Z_1 \cdots Z_{h-1} Z_h \cdots Z_\ell = a_1 \cdots a_{h-1} b_h \cdots b_\ell)} \\
&= \frac{\sum_{b_{h+1},\ldots,b_\ell \in \Sigma} P(W = a_1 \cdots a_i b_{h+1} \cdots b_\ell | X = Y)}{\sum_{b_h,\ldots,b_\ell \in \Sigma} P(W = a_1 \cdots a_{h-1} b_h \cdots b_\ell | X = Y)} \\
&= \frac{\sum_{b_{h+1},\ldots,b_\ell \in \Sigma} \frac{P(W = a_1 \cdots a_i b_{h+1} \cdots b_\ell)}{\cancel{P(X=Y)}}}{\sum_{b_h,\ldots,b_\ell \in \Sigma} \frac{P(W = a_1 \cdots a_{h-1} b_h \cdots b_\ell)}{\cancel{P(X=Y)}}}
\end{aligned}$$

$$\begin{aligned}
&= \frac{\sum_{b_{h+1},\ldots,b_\ell \in \Sigma} \cancel{P(W_1 \cdots W_{h-1} = a_1 \cdots a_{h-1})} P(W_h \cdots W_\ell = a_i b_{h+1} \cdots b_\ell) | W_{h-1} = a_{h-1})}{\sum_{b_h,\ldots,b_\ell \in \Sigma} \cancel{P(W_1 \cdots W_{h-1} = a_1 \cdots a_{h-1})} P(W_h \cdots W_\ell = b_h \cdots b_\ell | W_{h-1} = a_{h-1})} \\
&= P(W_h = a_h | W_{h-1} = a_{h-1}) \frac{\sum_{b_{h+1},\ldots,b_\ell \in \Sigma} P(W_{h+1} \cdots W_\ell = b_{h+1} \cdots b_\ell) | W_h = a_h)}{\sum_{b_h,\ldots,b_\ell \in \Sigma} P(W_h \cdots W_\ell = b_h \cdots b_\ell | W_{h-1} = a_{h-1})} \\
&= P(W_h = a_h | W_{h-1} = a_{h-1}) \frac{f(a_h, h)}{f(a_{h-1}, h-1)}.
\end{aligned}$$

On the other hand

$$
\begin{aligned}
P(Z_h = a_h | Z_{h-1} = a_{h-1}) &= \frac{\sum_{b_1 \cdots b_{h-2} b_{h+1} \cdots b_\ell} P(Z = b_1 \cdots b_{h-2} a_{h-1} a_i b_{h+1} \cdots b_\ell)}{\sum_{b_1 \cdots b_{h-2} b_h \cdots b_\ell} P(Z = b_1 \cdots b_{h-2} a_{h-1} b_h \cdots b_\ell)} \\
&= \frac{\sum_{b_1 \cdots b_{h-2} b_{h+1} \cdots b_\ell} P(W = b_1 \cdots b_{h-2} a_{h-1} a_i b_{h+1} \cdots b_\ell | \cancel{X = Y})}{\sum_{b_1 \cdots b_{h-2} b_h \cdots b_\ell} P(W = b_1 \cdots b_{h-2} a_{h-1} b_h \cdots b_\ell | \cancel{X = Y})} \\
&= \frac{\cancel{\sum_{b_1 \cdots b_{h-2}} P(W_1 \cdots W_{h-1} = b_1 \cdots b_{h-2} a_{h-1})} \sum_{b_{h+1} \cdots b_\ell} P(W_h \cdots W_\ell = a_i b_{h+1} \cdots b_\ell | W_{h-1} = a_{h-1})}{\cancel{\sum_{b_1 \cdots b_{h-2}} P(W_1 \cdots W_{h-1} = b_1 \cdots b_{h-2} a_{h-1})} \sum_{b_h \cdots b_\ell} P(W_h \cdots W_\ell = b_h \cdots b_\ell | W_{h-1} = a_{h-1})} \\
&= \frac{P(W_h = a_h | W_{h-1} = a_{h-1}) \sum_{b_{h+1} \cdots b_\ell} P(W_{h+1} \cdots W_\ell = b_{h+1} \cdots b_\ell | W_h = a_h)}{\sum_{b_h \cdots b_\ell} P(W_h \cdots W_\ell = b_h \cdots b_\ell | W_{h-1} = a_{h-1})} \\
&= P(W_h = a_h | W_{h-1} = a_{h-1}) \frac{f(a_h, h)}{f(a_{h-1}, h-1)}.
\end{aligned}
$$

□

Finally we take the “intersection-product” of Θ_α and Θ_β as constructed in Proposition 1 to define $E_{k_1 k_2 od}$ as

$$
E_{k_1 k_2 od}[ab, h] = \Theta_\alpha[ab, h] \cdot \Theta_\beta[ab, h] \cdot \frac{f(b, h)}{f(a, h-1)}, \qquad \text{(S10)}
$$

where $f(c, h)$ gives the probability of “Θ_α and Θ_β agree about symbols in positions after h on the condition they agree that the symbol in position h is c”.

Finally, the *deviation matrix* $\kappa_{k_1 k_2 od}$, defined as

$$
\kappa_{k_1 k_2 od} = \tau_{k_1 k_2 od} - E_{k_1 k_2 od}, \qquad \text{(S11)}
$$

gives the difference between observed and expected model. Deviation matrices for the PPM case will be visualized using a variant of the sequence logo in which positive values are shown above a separating line and negative values below it, see Fig. S3 for an example. For ADM models, the deviation model is visualised as a modification of the river-lake logo with blue arcs and states denoting positive values (observed value larger than expected) and red ones for negative values (observed value smaller than expected). See Fig. 2 for an example. Note also that the expected PPM and ADM of homodimers are always palindrome symmetric for orientations HH and TT.

Mixing parameters λ

Mixing parameters $\lambda = \{\lambda_k : k \in \{0\} \cup M \cup D^+ \cup D^-\}$ give the probability of each component of the mixture as follows:

λ_k, $k \in M$, is the probability that the sequence contains exactly one monomeric occurrence of motif θ_k and no other occurrences.

λ_k, $k = k_1k_2od \in D^+ \cup D^-$, is the probability that the sequence contains exactly one occurrence of motif $\tau_{k_1k_2od}$ and no occurrences of other motifs.

λ_0 is the probability that the sequence contains no motif occurrences.

For each pair (k_1, k_2), the array $(\lambda_{k_1k_2od})_{o \in \Omega_{k_1k_2}, d \in \Delta_{k_1k_2}}$ of mixing parameter values is called the *Co-Operative Binding table* (*COB table*) of motifs θ_{k_1} and θ_{k_2}. The values in a COB table indicate the orientation and spacing preferences of the dimeric structures that are composed of θ_{k_1} and θ_{k_2}.

Fig. 2 illustrates model η for binding motifs of TF LHX8.

S2 EM learning of the model

Given a training data set $X = \{X_1, X_2, \ldots, X_n\}$ consisting of n DNA sequences $X_i = X_{i1} \cdots X_{iL_i}$, where L_i is the length of the ith sequence, the EM algorithm (Dempster, Laird, and Rubin, 1977; Bailey and Elkan, 1995) is used for finding model parameters η which maximize the expectation of the likelihood $L(\eta|X,Z) = P(X,Z|\eta)$. Here latent variables Z give the 'missing information' used by the EM algorithm.

Latent variables are 0–1-valued random variables that indicate how the data X is aligned to the model. To align sequence X_i, there are latent variables $Z_{ik\cdot}$, $k \in \{0\} \cup M \cup D^+ \cup D^-$, with exactly one of them having value 1, that code the alignment as follows.

Case $Z_{i0} = 1$: Sequence X_i has no occurrences of motifs and is generated by the background model θ_0 alone.

Case $Z_{ikj} = 1$: If $k \in M$, then X_i has an occurrence of motif θ_k starting at position j. The rest of X_i is generated by the background model. If $k = k_1k_2od \in D^+ \cup D^-$ then X_i has an occurrence of motif τ_k at position j, that is, an occurrence of motif θ_{k_1} at position j and an occurrence of motif θ_{k_2} at $j + \ell_{k_1} + d$ such that the occurrences of θ_{k_1} and θ_{k_2} have relative orientation o.

We denote by S_{ik} the set of positions j at which motif k may occur in X_i. For $k \in M$ we have $S_{ik} = \{1, \ldots, L_i - \ell_k + 1\}$, and for $k = k_1k_2od \in D^+ \cup D^-$, $S_{ik} = S_{ik_1k_2od} = \{1, \ldots, L_i - (\ell_{k_1} + \ell_{k_2} + d) + 1\}$.

For notational convenience we make the following definitions. For PPM model $P(X_i, j, \theta_k) := \prod_{h=1}^{\ell_k} \theta_k[X_{i,j+h-1}, h]$ and similarly for ADM model $P(X_i, j, \theta_k) := \prod_{h=1}^{\ell_k} \theta_k[X_{i,j+h-2}, X_{i,j+h-1}, h]$.

The probability of X_i in model η, given the missing information $Z_{i\cdot}$, is straightforward to evaluate as follows. If sequence X_i contains no motif occurrences, i.e., $Z_{i0} = 1$, its probability is

$$P(X_i|Z_{i0} = 1, \eta) = \prod_{h=1}^{L_i} \theta_0[X_{ih}]. \tag{S12}$$

If X_i contains one motif occurrence, i.e., $Z_{ikj} = 1$ for some $k \in M$, $j \in S_{ik}$, its probability is

$$P(X_i|Z_{ikj} = 1, \eta) = P(X_i, j, \theta_k) \cdot \prod_{h \in B_1} \theta_0[X_{ih}], \tag{S13}$$

where $B_1 = \{1, \ldots, L_i\} \setminus [j, j + \ell_k)$.

For the dimeric binding we have two cases: independent ($d \geq \delta$) and dependent ($d < \delta$). Let first $k = k_1 k_2 od \in D^+$. Define the set $B_2 = \{1, \ldots, L_i\} \setminus \big([j, j + \ell_{k_1}) \cup [j + \ell_{k_1} + d, j + (\ell_{k_1} + \ell_{k_2} + d))\big)$. Then the probability of X_i is

$$P(X_i|Z_{ikj} = 1, \eta) = P(X_i, j, \theta_{k_1}^{o_1}) \cdot P(X_i, j + \ell_{k_1} + d, \theta_{k_2}^{o_2}) \cdot \prod_{h \in B_2} \theta_0[X_{ih}]. \tag{S14}$$

Let then $k = k_1 k_2 od \in D^-$. The probability of X_i is

$$P(X_i|Z_{ikj} = 1, \eta) = P(X_i, j, \tau_{k_1 k_2 od}) \cdot \prod_{h \in B_2} \theta_0[X_{ih}]. \tag{S15}$$

Now the joint likelihood of the model parameters, given data X and missing information Z, is the product of mixture probabilities of each X_i:

$$L(\eta|X,Z) = P(X,Z|\eta) = \prod_{i=1}^{n} \Big(Z_{i0} \cdot \lambda_0 \cdot P(X_i|Z_{i0} = 1, \eta) + \sum_{k \in M \cup D^+ \cup D^-} \sum_{j \in S_{ik}} Z_{ikj} \cdot \frac{\lambda_k}{|S_{ik}|} \cdot P(X_i|Z_{ikj} = 1, \eta)\Big).$$

Note that, to simplify notation, we have ignored the fact that we should consider motif occurrences appearing in the reverse DNA strand as well. For this algorithm to work in the two-stranded case, a new index should be added, which specifies the direction ($+1$ or -1) of a monomer or a dimer occurrence. Then in all the places where we sum over j, we should sum over the directions as well. Moreover, to make the probabilities to add up to one, an additional division by two should be performed where we currently divide by $|S_{ik}|$.

As for each i, exactly one of the latent values $Z_{i\cdot}$ equals 1 and the others are zeros, the

log-likelihood has the following form:

$$\log P(X,Z|\eta) = \sum_{i=1}^{n} \Big[Z_{i0} \log\big(\lambda_0 P(X_i|Z_{i0}=1,\eta)\big) + \sum_{\substack{k\in M\cup D^+\cup D^- \\ j\in S_{ik}}} Z_{ikj} \log\Big(\frac{\lambda_k}{|S_{ik}|} P(X_i|Z_{ikj}=1,\eta)\Big)\Big]. \quad \text{(S16)}$$

The EM algorithm repeatedly applies the following rule to update $\eta = (\theta, \psi, \lambda)$ until convergence:

$$\eta^{(t+1)} := \arg\max_{\eta} E_{Z|X,\eta^{(t)}} \log P(X,Z|\eta).$$

One iteration of the algorithm, indexed with t, consists of an E-step and an M-step. These steps are described next.

Expectation step

E-step finds the expectation of log-likelihood (S16) for current parameter values $\eta^{(t)}$. By linearity of expectation, this reduces to finding the expected values $z_{i\cdot}$ of latent variables $Z_{i\cdot}$. By noting that 0 and 1 are the only possible values of a latent variable, and by applying the Bayes rule, one can see that the expected values and hence the update rule of the E-step becomes, for $k \in \{0\} \cup M \cup D^+ \cup D^-$ and $j \in S_{ik}$, as follows

$$z_{i0}^{(t)} := E[Z_{i0}|X,\eta^{(t)}] = P(Z_{i0}=1|X_i,\eta^{(t)}) = \frac{\lambda_0^{(t)} \cdot P(X_i|Z_{i0}=1,\eta^{(t)})}{P(X_i|\eta^{(t)})}, \quad \text{(S17)}$$

$$z_{ikj}^{(t)} := E[Z_{ikj}|X,\eta^{(t)}] = P(Z_{ikj}=1|X_i,\eta^{(t)}) = \frac{\lambda_k^{(t)}/|S_{ik}| \cdot P(X_i|Z_{ikj}=1,\eta^{(t)})}{P(X_i|\eta^{(t)})}. \quad \text{(S18)}$$

Here probability $P(X_i|Z_{i0}=1,\eta^{(t)})$ is given by (S12) and probability $P(X_i|Z_{ikj}=1,\eta^{(t)})$ by (S13), (S14), or (S15), and

$$P(X_i|\eta^{(t)}) = \lambda_0^{(t)} \cdot P(X_i|Z_{i0}=1,\eta^{(t)}) + \sum_{\substack{k\in M\cup D^+\cup D^- \\ j\in S_{ik}}} \lambda_k^{(t)}/|S_{ik}| \cdot P(X_i|Z_{ikj}=1,\eta^{(t)}). \quad \text{(S19)}$$

Maximization step

M-step maximizes the expectation of log-likelihood for current $z^{(t)}$ by updating parameters $\eta = (\theta, \psi, \lambda)$. The form of log-likelihood (S16) is such that the M-step is of Baum–Welch type: parameters are updated by normalizing the expected counts of using different components of the model when X is aligned to the model according to $z^{(t)}$.

The update rules for mixing parameters become:

$$\lambda_0^{(t+1)} := \frac{1}{n}\sum_{i=1}^{n} z_{i0}^{(t)}, \text{ and} \tag{S20}$$

$$\lambda_k^{(t+1)} := \frac{1}{n}\sum_{i=1}^{n}\sum_{j\in S_{ik}} z_{ikj}^{(t)}, \; k \in M \cup D^{+} \cup D^{-}. \tag{S21}$$

To update θ and ψ we first accumulate the expected counts of how many times each mixture component is used when X is aligned with $\eta^{(t)}$. For all $k \in M$, we get the $H \times \ell_k$ matrices of expected counts of the monomer motifs as

$$W_k = \sum_{i=1}^{n}\Big[\sum_{j\in S_{ik}} z_{ikj}^{(t)} I_{\ell_k}(i,j) + \sum_{kk'od\in D^{+}}\sum_{j\in S_{ikk'od}} z_{ikk'odj}^{(t)} I_{\ell_k}^{o_1}(i,j) + \sum_{k'kod\in D^{+}}\sum_{j\in S_{ik'kod}} z_{ik'kodj}^{(t)} I_{\ell_k}^{o_2}(i,j+\ell_{k'}+d)\Big].$$

Here $I_{\ell_k}(i,j)$ is a $H \times \ell_k$ matrix-valued indicator function such that for PPM models we set

$$I_{\ell_k}(i,j)[a,h] = \begin{cases} 1 & \text{if } X_i[j+h-1] = a, \\ 0 & \text{otherwise.} \end{cases} \tag{S22}$$

For ADM models, the $H \times \ell_k$ matrix-valued indicator function, $H = 16$, is defined as

$$I_{\ell_k}(i,j)[ab,h] = \begin{cases} 1 & \text{if } h > 1 \text{ and } X_i[j+h-2 : j+h-1] = ab \\ 1 & \text{if } h = 1, X_i[j+h-1] = b \text{ and } a = \mathrm{A} \\ 0 & \text{otherwise.} \end{cases} \tag{S23}$$

Again, $I_{\ell_k}^{-1}(i,j)$ is the reverse complement of $I_{\ell_k}(i,j)$. Note that the above aggregation of W_k implements the modularity of binding: a monomer model θ_k gets its counts from monomeric occurrences of θ_k as well as from occurrences of θ_k as an independent component of a dimer. Since the monomer models are not learned from the overlapping cases, there is no coupling

between the monomers and the deviation matrices, i.e. both are uniquely defined.

For $k \in D^-$, the $H \times (\ell_{k_1} + \ell_{k_2} + d)$ matrix of the expected counts is

$$W_k = W_{k_1 k_2 od} = \sum_{i=1}^{n} \sum_{j \in S_{ik_1 k_2 od}} z_{ik_1 k_2 od j}^{(t)} I_{\ell_{k_1}+\ell_{k_2}+d}(i,j). \tag{S24}$$

According to our modularity constraint the columns of $W_{k_1 k_2 od}$ that are outside the bridging segment should be modeled with θ_{k_1} and θ_{k_2}. They are therefore added to W_{k_1} and W_{k_2} as follows

$$W_{k_1}^{o_1}[\cdot, 1 : \ell_{k_1} + \min(d,0) - 1] \mathrel{+}= W_{k_1 k_2 od}[\cdot, 1 : \ell_{k_1} + \min(d,0) - 1], \tag{S25}$$

$$W_{k_2}^{o_2}[\cdot, \max(-d,0) + 2 : \ell_{k_2}] \mathrel{+}= W_{k_1 k_2 od}[\cdot, \ell_{k_1} + \max(d,0) + 2 : \ell_{k_1} + \ell_{k_2} + d]. \tag{S26}$$

The count vector of the background model is obtained as

$$\begin{aligned} W_0 = Q_X &- \sum_{k \in M} \sum_{h=1}^{\ell_k} W_k[\cdot, h] \\ &- \sum_{k_1 k_2 od \in D^-} \sum_{h=\ell_{k_1}+\min(d,0)}^{\ell_{k_1}+1+\max(d,0)} W_{k_1 k_2 od}[\cdot, h], \end{aligned}$$

where $Q_X = [Q_X^{\mathrm{A}}, Q_X^{\mathrm{C}}, Q_X^{\mathrm{G}}, Q_X^{\mathrm{T}}]^T$ is the column-vector of total counts of alphabet symbols in the data set X.

Pseudocounts are added to count matrices W_k such that in case of PPM, the 4×1 pseudocount vector $0.01 \cdot \theta_0$ is added to each column of the matrices, and in case of ADM, the 16×1 pseudocount vector $(0.01 \cdot \theta_0[a] \cdot \theta_0[b])_{a,b \in \Sigma}$ is added to each column of the matrices. When normalized column-wise, the matrices W_k give updated θ_k for $k \in \{0\} \cup M$:

$$\theta_0^{(t+1)}[\cdot] := \frac{W_0[\cdot]}{\sum_{b \in \Sigma} W_0[b]}, \tag{S27}$$

$$\theta_k^{(t+1)}[\cdot, h] := \frac{W_k[\cdot, h]}{\sum_{b \in \Sigma} W_k[b, h]}, \text{ for PPM} \tag{S28}$$

and, in case of ADM, for all $a \in \Sigma$

$$\theta_k^{(t+1)}[a\cdot, h] := \begin{cases} \frac{W_k[a\cdot,h]}{\sum_{b \in \Sigma} W_k[ab,h]} & \text{if } 1 < h \leq \ell_k, \\ \frac{W_k[\mathrm{A}\cdot,1]}{\sum_{b \in \Sigma} W_k[\mathrm{A}b,1]} & \text{if } h = 1. \end{cases} \tag{S29}$$

Note that for the ADM model the initial probabilities were stored four times.

Similarly, the bridging segments of W_k, $k = k_1 k_2 od \in D^-$, give updated bridging PPMs

ψ_k:

$$\psi^{(t+1)}_{k_1k_2od}[\cdot,h] := \frac{W_{k_1k_2od}[\cdot,h+\ell_{k_1}+\min(d,0)-1]}{\sum_{b\in\Sigma} W_{k_1k_2od}[b,h+\ell_{k_1}+\min(d,0)-1]}, \tag{S30}$$

where $h = 1,\dots,|d|+2$. And for ADMs

$$\psi^{(t+1)}_{k_1k_2od}[a\cdot,h] := \begin{cases} \frac{W_{k_1k_2od}[a\cdot,h+\ell_{k_1}+\min(d,0)-1]}{\sum_{b\in\Sigma} W_{k_1k_2od}[ab,h+\ell_{k_1}+\min(d,0)-1]} & \text{for } h = 2,\dots,|d|+2, \\ \frac{W_{k_1k_2od}[\mathrm{A}\cdot,h+\ell_{k_1}+\min(d,0)-1]}{\sum_{b\in\Sigma} W_{k_1k_2od}[\mathrm{A}b,h+\ell_{k_1}+\min(d,0)-1]} & \text{for } h = 1. \end{cases} \tag{S31}$$

S3 Details of implementation

EM iterations

As the EM algorithm converges to a local optimum, it is crucial to use good initial values for the parameters. Initial models $\theta^{(1)}_1,\dots,\theta^{(1)}_p$ are obtained from input data X and seeds $s_1,\dots,s_p$ using the *multinomial method* (Jolma, Kivioja, Toivonen, et al., 2010). Initial bridging models $\psi^{(1)}_{k_1k_2od}$ are obtained from input data X and *combined seeds* $s^{(1)}_{k_1k_2od}$ using the multinomial method. A combined seed is constructed by orienting seeds s_{k_1} and s_{k_2} according to orientation o, spacing them by d symbols, and replacing the symbols in the bridging segment by the neutral IUPAC symbol `N`. This gives sequence y. Then the combined seed $s^{(1)}_{k_1k_2od}$ is the highest counting non-palindromic subsequence of input data X that matches with y. A non-palindromic seed makes it possible for the EM search to break the symmetry and find non-palindromic models. Background model is initialized as $\theta^{(1)}_0 := Q_X/|Q_X|$ where $Q_X = [Q^{\mathrm{A}}_X, Q^{\mathrm{C}}_X, Q^{\mathrm{G}}_X, Q^{\mathrm{T}}_X]^T$ is the column-vector of total counts of alphabet symbols in X. The mixing parameters $\lambda^{(1)}$ are initialized as follows:

$$\lambda^{(1)}_0 := 0.5,$$

$$\lambda^{(1)}_k := \begin{cases} 0.3/p & \text{if } R \text{ is non-empty and} \\ 0.5/p & \text{otherwise, for all } k \in \{1,\dots,p\}, \end{cases}$$

$\lambda^{(1)}_{k_1k_2} := 0.2/|R|$, for all $(k_1,k_2) \in R$. Within a COB table the value $0.2/|R|$ is divided evenly among the cells as $\lambda^{(1)}_{k_2k_2od} := \lambda^{(1)}_{k_1k_2}/(|\Omega_{k_1k_2}|\cdot|\Delta_{k_1k_2}|)$.

The EM iterations then proceed as follows:

$t := 0$.
Repeat
  $t := t + 1$
  Compute $z^{(t)}$ using Eqs. (S17) and (S18)
  Compute $\lambda^{(t+1)}$ using Eqs. (S21) and (S20)
  Compute $\theta^{(t+1)}$ using Eqs. (S27) and (S28) or (S29)
  Compute $\psi^{(t+1)}$ using Eq. (S30) or (S31)
until $t = \texttt{maxiter}$ or $|(\theta^{(t+1)}, \psi^{(t+1)}) - (\theta^{(t)}, \psi^{(t)})| < \varepsilon$.
Output $(\theta^{(t+1)}, \lambda^{(t+1)}, \psi^{(t+1)}, \kappa^{(t+1)})$

It should be noted that the above algorithm outputs the deviation matrix κ just for completeness. As κ is a derived parameter, it could be evaluated from θ and ψ in a post-processing phase as well, using Eqs. S1,S2,S8,S10,S11.

Pruning the search

MODER2 implementation makes some heuristic modifications to the EM framework of Section 3 in order to speed-up the search and to utilize prior knowledge of data quality.

First, any dimeric component k whose λ_k gets below a small threshold (default 0.001) is eliminated as k is too weak. This is done by setting $\lambda_k := 0$. Blank entries of COB tables indicate eliminated dimers. Similarly, as the information content of well-known binding affinity models is on average quite high while low information content may indicate contamination from background, MODER2 trims during the EM all overlapping (i.e., $d < 0$) dimeric mixture components k whose average column-wise information content in the overlapping area goes below a threshold (default 0.40 bits).

Second, MODER2 learns new values $\theta_1^{(t+1)}, \ldots, \theta_p^{(t+1)}$ of monomeric models from monomeric occurrences of the monomer as well as from dimeric occurrences of the monomer such that the distance d between the components is large enough (default $d \geq \delta = 4$). This is because such isolated occurrences within a dimer are supposed to give the best data for a monomer model, not distorted by close-by other sites such as the other component of a dimer.

Limiting the search to Hamming neighbourhood

The third modification is motivated by the fact that transcription factors may have different binding motifs whose consensus sequences are only a few Hamming steps apart. To minimize disturbance from such similar motifs and from background, MODER2 restricts the learning

of models $\theta_k^{(t+1)}$ and $\psi_k^{(t+1)}$ to high-affinity training sequences. Such sequences are identified by the heuristic rule that their Hamming distance to the consensus sequences (sequences with highest probability of the models found so far) is small, at most ρ. Monomer model $\theta_k^{(t+1)}$ is learned from data sequences that are in the ρ-Hamming neighborhood of the seed (using the consensus sequence as the seed) of $\theta_k^{(t)}$, where by default $\rho = 2$ for PPM models and $\rho = 3$ for ADM models. Bridging model $\psi_{k_1k_2od}^{(t+1)}$ is learned from data sequences that are in the ρ-Hamming neighborhood of combined seed $s_{k_1k_2od}^{(t)}$. The combined seed is obtained as the initial combined seed $s_{k_1k_2od}^{(1)}$ (see Section EM Iterations) but using the seeds of $\theta_{k_1}^{(t)}$ and $\theta_{k_2}^{(t)}$. MODER2 uses this seed-guided EM search by default, with the standard search as an option.

Restriction to sequences that belong to a Hamming neighborhood of the seed gives a biased sample (see (Jolma, Kivioja, Toivonen, et al., 2010; Toivonen, Taipale, and Ukkonen, 2017)) that does not directly follow the count distribution of full sample. To fix this seed bias, the maximization phase of the EM algorithm needs a modification as follows. For the PPM case, we only need to change the indicator function I that is utilized in accumulating the expected counts (Eq. (S22)). Let's denote the current site by $x = X_i[j : j+\ell-1]$. The new indicator function J to accumulate the counts correctly is

$$J_\ell(i,j)[a,h] = \begin{cases} 1 & \text{if } x_h = a \text{ and } \mathscr{H}(x,s) - [x_h \neq s_h] \leq \rho - 1 \\ 0 & \text{otherwise,} \end{cases} \tag{S32}$$

where $\mathscr{H}(\cdot,\cdot)$ is the Hamming distance.

In the ADM case the seed bias correction is more complicated. We want to learn an ADM θ of length ℓ that represents a target distribution P which is assumed first-order Markov. Assume that we have counts $\omega[x]$ for every $x \in \Sigma^\ell$ such that $\omega[x] \propto P(x)$. Then $\theta[ab,h]$ could be estimated as

$$P(X_h = b | X_{h-1} = a) \propto \theta[ab,h] := \sum_{x\in\Sigma^\ell[ab,h]} \omega[x] / \sum_{x\in\Sigma^\ell[a\cdot,h]} \omega[x] \tag{S33}$$

where $\Sigma^\ell[ab,h] = \{x \in \Sigma^\ell | x_{h-1}x_h = ab\}$. However, if limited to ρ-Hamming neighborhood $\mathscr{H}_\rho(s)$ of seed s, the training sample is in $\Sigma^\ell \cap \mathscr{H}_\rho(s)$ instead of full Σ^ℓ, i.e., the sample has seed bias which we need to fix.

To derive bias correction for the above learning rule of $\theta[ab,h]$, let $a \in \Sigma$ and $1 \leq h \leq \ell$. For every $0 \leq r \leq \rho - 1$ define $U_r := \mathscr{H}_{\rho-r-1}(s_{h+1:\ell}) \subset \Sigma^{\ell-h}$. For any v such that $r := \mathscr{H}(va, s_{1:h-1}) \leq \rho - 1$ and $b \in \Sigma$ define counts $\omega[vab,h] := \sum_{u\in U_r} \omega[vabu]$. Note that all such

sequences $vabu$ are in $\mathscr{H}_\rho(s)$. Further define

$$\omega^{ab,h}(r) := \sum_{\mathscr{H}(va,s_{1:h-1})=r} \omega[vab,h].$$

Proposition 2. *The probability of observing b in position h of the motif on the condition that the previous symbol was a is*

$$P_h(b|a) \approx \frac{\sum_{r=0}^{\rho-1} \frac{\omega^{ab,h}(r)}{P(U_r|b)}}{\sum_{c\in\Sigma}\sum_{r=0}^{\rho-1} \frac{\omega^{ac,h}(r)}{P(U_r|c)}}.$$

Proof. Fix v such that $r := \mathscr{H}(va,s_{1:h-1}) \leq \rho-1$. Now $\omega[vab,h] \propto P(vabU_r) = P(va)P(bU_r|a)$, for all $b \in \Sigma$, which implies that $\omega[vab,h] \propto p(bU_r|a)$, for all $b \in \Sigma$. Clearly $\omega^{ab,h}(r) \propto P(bU_r|a)$. On the other hand, from $P(bU_r|a) = P_h(b|a)P(U_r|b)$ we get $P_h(b|a) = \frac{P(bU_r|a)}{P(U_r|b)}$. It follows that $P_h(b|a) \propto \frac{\omega^{ab,h}(r)}{P(U_r|b)}$. Because the above holds for all $0 \leq r \leq \rho-1$, we get $P_h(b|a) \propto \sum_{r=0}^{\rho-1} \frac{\omega^{ab,h}(r)}{P(U_r|b)}$. From this the claim follows after normalization. □

The correction factor $1/P(U_r|b)$ of Proposition 2 depends on P, which we are trying to learn in the end. This is not a problem, however, since we can use the segment of θ learned so far in the role of P: start computing the columns of matrix θ from the right-most column and proceed towards the left-most column. Denote the learned probability of the sequences in the r'-neighbourhood of $s_{h+1:\ell}$, where $0 \leq r' \leq \rho-1$, with condition that the previous symbol is b, as

$$\gamma^{b,h}(r') := \sum_{w\in\mathscr{H}_{r'}(s_{h+1:\ell})} \theta[bw_1,h+1]\theta[w_1w_2,h+2]\cdots\theta[w_{\ell-h-1}w_{\ell-h},\ell].$$

Then $P(U_r|b) \propto \gamma^{b,h}(\rho-r-1)$. Since $\gamma^{b,\ell}(r') = 1$ for all $b \in \Sigma$ and $r' \geq 0$, we get the right-most column of θ easily. Once the correction has been done for column h, the column can be normalised in the usual way to obtain $\theta[\cdot,h]$, which can subsequently be used to compute the correction for column $h-1$.

The indicator function to accumulate the (expected) counts in a Hamming neighbourhood becomes as follows. Let again $x = X_i[j : j+\ell-1]$ be the current site and define $H \times \ell \times \rho$ matrix-valued indicator function $J_\ell(i,j)$ as

$$J_\ell(i,j)[ab,h,r] = \begin{cases} 1 & \text{if } h>1, x_{h-1:h}=ab, \mathscr{H}(x,s)-[x_h \neq s_h] \leq \rho-1 \text{ and } \mathscr{H}(x_{1:h-1},s_{1:h-1})=r \\ 1 & \text{if } h=1, r=0, x_h=b, a=\mathrm{A} \text{ and } \mathscr{H}(x,s)-[x_h\neq s_h] \leq \rho-1 \\ 0 & \text{otherwise.} \end{cases} \tag{S34}$$

Using indicator function J (as I is used in Eq. (S24)) we get weight matrices $(\omega^{ab,h}(r))$ that have shape $H \times \ell \times \rho$. The seed bias can then be corrected as follows:

$$W[ab,h] = \sum_{r=0}^{\min(\rho,h)-1} \omega^{ab,h}(r)/\gamma^{b,h}(\min(\rho - r - 1, \ell - h)),$$

to get counts to be used in Eq. (S29). This correction depends on θ, which we are trying to compute but, as explained above, by evaluating the columns of θ from right to left, the columns that are needed for the correction are ready in due time. When the dinucleotide counts ω^{ab} are very small, the division in the seed bias correction becomes sensitive to sampling error. Therefore we add to the divisors $\gamma^{b,h}$ a damping term which is proportional to $\omega_{\min}^{-1/2}$, where $\omega_{\min}$ is the smallest dinucleotide count used in the derivation of dinucleotide probabilities θ^{ab} that occur beyond column h.

The derived method for ADM learning in Hamming neighbourhood is given as Algorithm S1.

Algorithm S1: AlignADM — ADM learning in Hamming neighbourhood

input : motif length ℓ, IUPAC seed $S = S_1 \cdots S_\ell$, Hamming radius $\rho \geq 2$, weights (or counts) for sequences in $\mathscr{H}_\rho(S)$

output : First order Markov chain $\theta^{ab,h}$ where $a \in \Sigma$ and $b \in \Sigma$, $1 \leq h \leq \ell$

for $h \leftarrow 1$ **to** ℓ, $r \leftarrow 0$ **to** $\rho - 1$, $ab \in \Sigma^2$ **do**
$\omega^{ab,h}(r) \leftarrow 0$;
for $u \in \mathscr{H}_\rho(S)$ **do**
$\omega(u) \leftarrow weight(u) + 0.01 * \theta_0(u)$; `// Add pseudocount to weights`
$r \leftarrow 0$;
for $h \leftarrow 1$ **to** ℓ **do**
if $u_h \notin S_h$ **or** $h(u,S) \leq \rho - 1$ **then**
$\omega^{u_{h-1}u_h,h}(r) \leftarrow \omega^{u_{h-1}u_h,h}(r) + \omega(u)$;
if $u_h \notin S_h$ **then**
$r \leftarrow r + 1$;

for $h \leftarrow \ell$ **to** 1 **do**
for $b \in \Sigma$ **do** `// Initialize out-of-bounds values`
$\gamma^{b,h}(-1) \leftarrow 0$;
$\gamma^{b,h}(\ell - h + 1) \leftarrow 0$;
for $r' \leftarrow 0$ **to** $\min(\rho - 1, \ell - h)$ **do** `// Compute correction factors`
if $h = \ell$ **then**
for $b \in \Sigma$ **do** $\gamma^{b,h}(r') \leftarrow 1$;
else
for $b \in \Sigma$ **do**
$\gamma^{b,h}(r') \leftarrow \sum_{c \in \Sigma} \theta^{bc,h+1} \cdot \gamma^{c,h+1}(r' - [c \notin S_{h+1}])$;
for $ab \in \Sigma_{h-1} \times \Sigma$ **do** `// Correct for seed bias`
$W^{ab,h} \leftarrow 0$;
for $r \leftarrow 0$ **to** $\min(\rho - 1, h - 1)$ **do**
$W^{ab,h} \leftarrow W^{ab,h} + \omega^{ab,h}(r) / \gamma^{b,h}(\min(\rho - r - 1, \ell - h))$;
for $a \in \Sigma_{h-1}$ **do** `// Normalize`
for $b \in \Sigma$ **do**
$\theta^{ab,h} \leftarrow W^{ab,h} / \sum_{c \in \Sigma} W^{ac,h}$;

Omitting seed bias correction

In our experiments the seed bias correction was observed to have a relatively small effect on the learning result. A reason for this could be that the model bias (i.e., the inability of the model family to represent accurately the affinity distributions of TF binding motifs) may dominate. Omitting seed bias correction means that we avoid the noise sensitive division by the correction factor.

If seed bias correction is omitted, the division by the correction factor is not needed, and the indicator function $J_\ell(i,j)$ with $H \times \ell$ matrix values becomes:

$$J'_\ell(i,j)[ab,h] = \begin{cases} 1 & \text{if } x_{h-1:h} = ab, \mathscr{H}(x,s) - [x_h \neq s_h] \leq \rho - 1 \text{ and } 1 < h \leq \ell_k, \\ 1 & \text{if } x_h = b, a = \text{A}, \mathscr{H}(x,s) - [x_h \neq s_h] \leq \rho - 1 \text{ and } h = 1, \\ 0 & \text{otherwise.} \end{cases} \tag{S35}$$

The normalization of the expected counts is handled as before in Eq. S29.

S4 Sanity checks with generated data

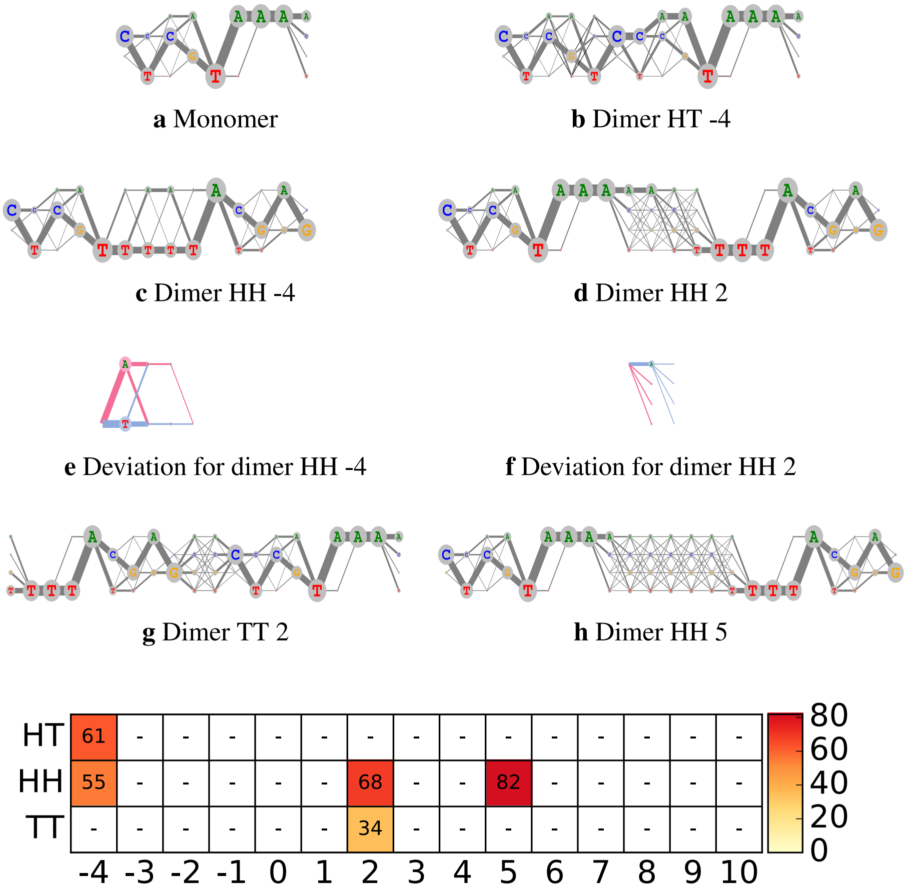

a Monomer **b** Dimer HT -4

c Dimer HH -4 **d** Dimer HH 2

e Deviation for dimer HH -4 **f** Deviation for dimer HH 2

g Dimer TT 2 **h** Dimer HH 5

i Homodimeric COB table

Figure S1: **Mixture of ADMs for generated data.** **(a)** Monomeric ADM of HOXB13 originally learned from SELEX data using MODER2. **(b)**, **(c)**, **(d)**, **(g)**, **(h)** Using the monomeric model we created the dimeric ADMs HT -4, HH -4, HH 2, TT 2, and HH 5. The dimeric models were the expected models (see Supplementary Section S1) with the exception of dimers HH -4 and HH 2 which were slightly perturbed from the expectation. **(e)**, **(f)** The deviation from the expected models for dimers HH -4 and HH 2. In the experiments we used three values for the total signal fraction: 0.03, 0.30, and 0.90. **(i)** The homodimeric COB table when the total signal fraction is 0.30. The λ-values for each model component are: the uniform background ($\lambda = 0.70$), ADM for monomer ($\lambda = 0$), ADMs for homodimers HT -4 ($\lambda = 0.061$), HH -4 (0.055), HH 2 (0.068), TT 2 (0.034), and HH 5 (0.082). Using this model, we generated 100 000 sequences of length 40 bp.

Table S2: **Dependency on Hamming radius.**

ρ λ	2	3	4	5	6	7	8	9	∞
0.03	0.226	0.150	0.108	0.480	0.174	0.556	0.110	**0.097**	0.645
0.30	0.175	0.188	0.157	0.149	0.175	0.172	**0.051**	0.158	0.161
0.90	0.174	0.163	0.152	0.151	0.150	**0.147**	0.148	**0.147**	**0.147**

Distances between re-learned model and the original one, shown in Fig. S1, for different values of Hamming radius ρ and total signal fraction λ. The distance measures used were the weighted max norm for ADM models, and max norm for the lambda values of each model component.

Table S3: **Seeds used in the experiment shown in Figure S2.**

Factor 1	Factor 2	Seed 1	Seed 2	Hamming distance
ARNTL	MLXIPL	GTCACGTGAC	ATCACGTGAT	2
MSC	MLXIPL	AACAGCTGTT	ATCACGTGAT	4
MSC	NHLH1	AACAGCTGTT	CGCAGCTGCG	4
BHLHE41	MLXIPL	GTCACGTGAC	ATCACGTGAT	2
FLI1	CEBPG	ACCGGAARYN	NTTRCGCAAY	8.25

In case of IUPAC characters, the Hamming distance between two characters is one minus the Jaccard index between the sets defined by the characters.

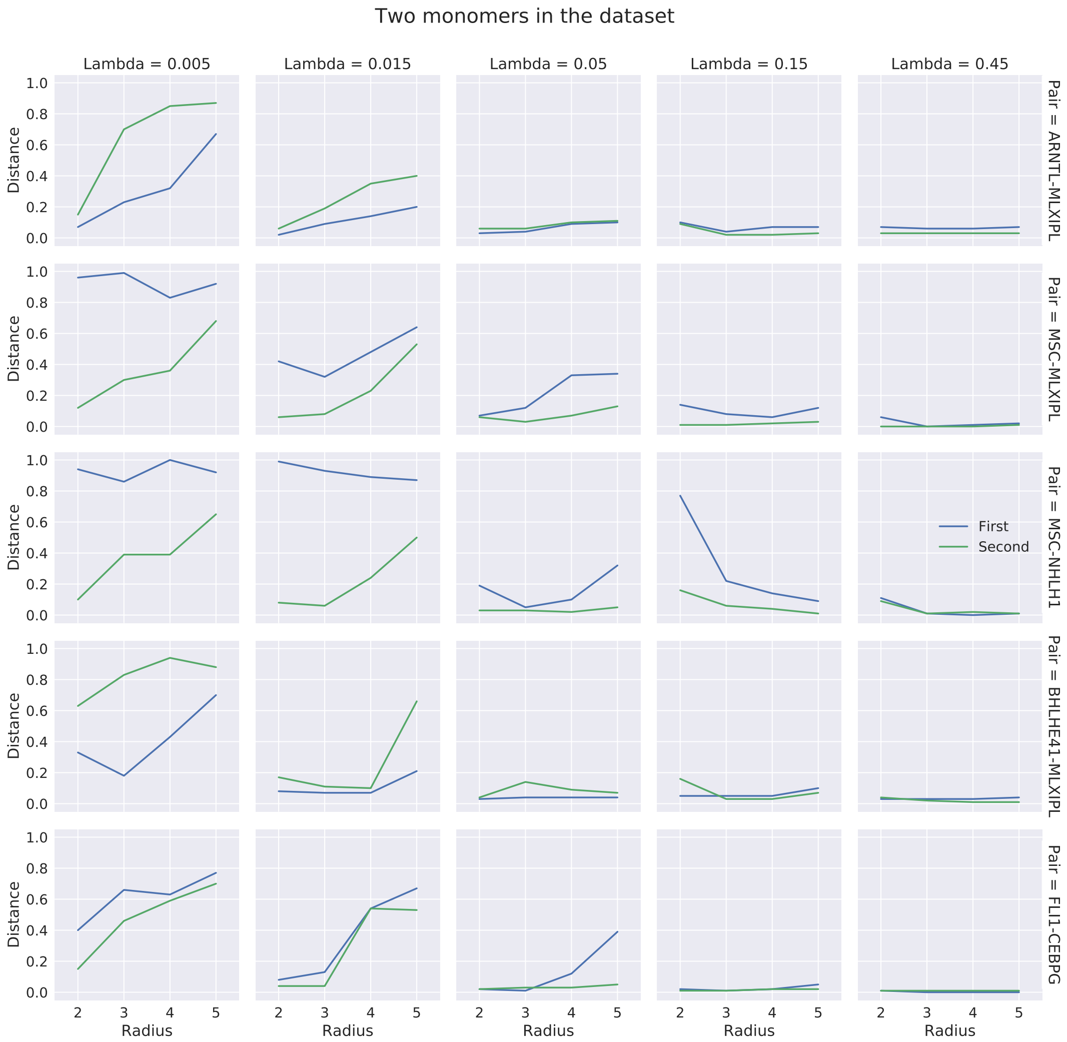

Figure S2: From a set of ADM models learned from SELEX data, we collected four pairs of transcription factors where each pair had non-IUPAC consensus sequences at Hamming distance 2 or 4 apart from each other. A fifth TF pair was selected to have longer distance between their consensus sequences that also included IUPAC codes. The consensus sequences used for each factor are shown in Table S3. Then with these models we generated for each TF pair five data sets (n=100 000, L=40) with fraction of a TF λ being either 0.005, 0.015, 0.05, 0.15, and 0.45, i.e., fraction λ of generated sequences contained a binding site of the first factor and same fraction contained a binding site of the second factor. The rest of the positions were filled with uniform random distribution of nucleotides. The distances between the generating models and the models relearned from the generated data are measured using the weighted max norm. The results show that for low signal fractions, small Hamming radius (2 or 3) gives the most accurate results.

S5 Order-zero vs order-one motifs of HNF4A and ARGFX

Evaluation and visualization of correlation R^2

The quality of models is measured using correlation (R^2) between occurrence counts and model scores of 8-mers or 10-mers of HT-SELEX data. When counting the k–mers, all occurrences and both directions were considered. As the score of a k-mer x by a single ADM or PPM θ we used the maximum value of $\log \frac{\theta'(y)}{\theta_0(y)}$ when y and θ' go over all intersections of θ and x and of θ and reverse complement $\bar{x}$. As the score of x by a mixture of ADMs or PPMs $\theta_1, \ldots, \theta_t$, whose mixing parameters are $\lambda_1, \ldots, \lambda_t$, we used $\lambda_1 S_1 + \cdots + \lambda_t S_t$ where $S_1, \ldots, S_t$ are the individual scores of x by the ADMs or PPMs. The scatter plots in the figures visualize the counts and scores of different 8-mers in hexagonal bins. The gray-scale color of a bin reflects the number of different k–mers in that bin, with a darker color meaning higher number of different k–mers. As the early cycles of SELEX data can contain large proportion of nonspecific sequences (background), the counts were corrected against background using the data of the previous SELEX cycle, as described in (Jolma, Kivioja, Toivonen, et al., 2010).

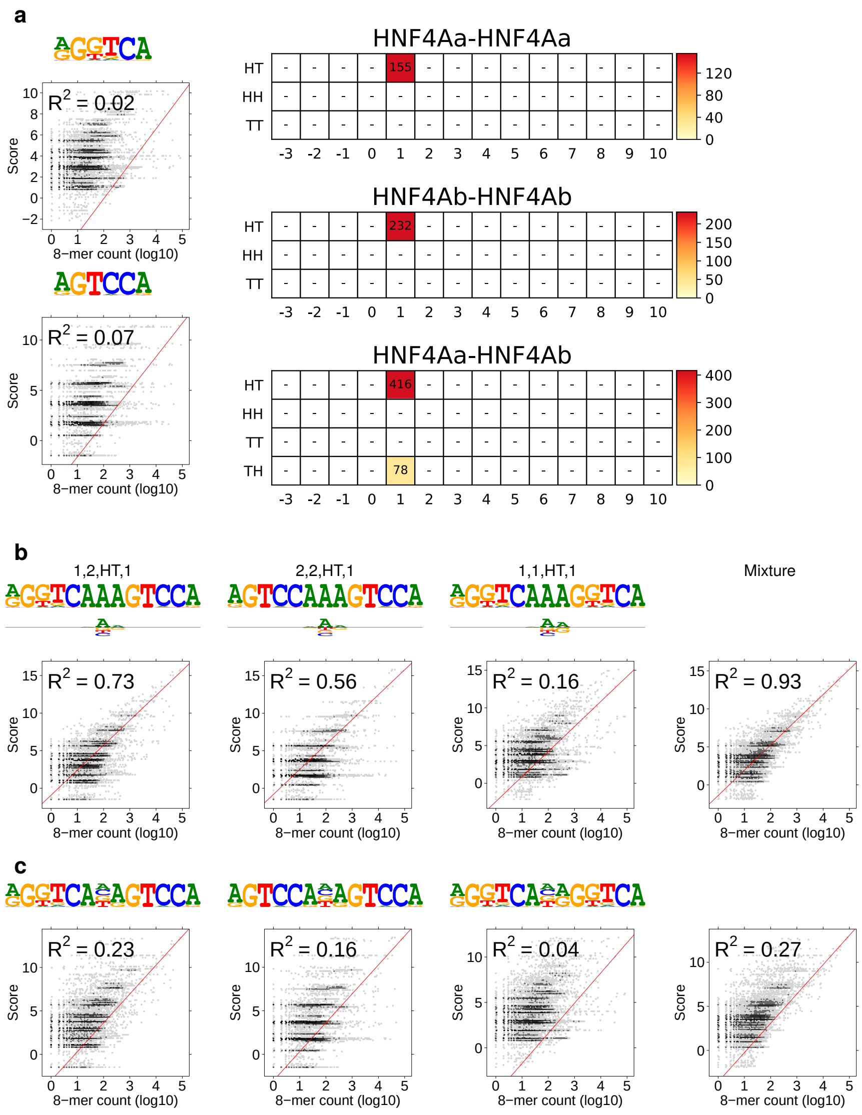

Figure S3: **Modularity analysis of HNF4A using two monomers, PPM case. (a)** Monomer PPMs θ_1 and θ_2 ($\lambda_1 = 0.016, \lambda_2 = 0.008$) and the COB tables in units of integer multiples of 0.001, as learned from data by MODER. **(b)** Three dimeric PPMs $\tau_{1,2,\mathrm{HT},1}$, $\tau_{2,2,\mathrm{HT},1}$, and $\tau_{1,1,\mathrm{HT},1}$ ($\lambda = 0.416, 0.232, 0.155$) alone cover about 85% of the signal and were included in the final mixture by the 85% rule. Deviations are depicted below the PPM logos. The mixture has much higher correlation than any individual PPM. **(c)** Correlation analysis as in **b** but for the PPMs $E_{1,2,\mathrm{HT},1}$, $E_{2,2,\mathrm{HT},1}$, and $E_{1,1,\mathrm{HT},1}$, that are expected under the independence assumption. All R^2-values for the learned and expected PPMs differ remarkably, reflecting the large deviations between the learned and the expected PPMs. The expected model does not detect the `AAA` sequence connecting the half-sites.

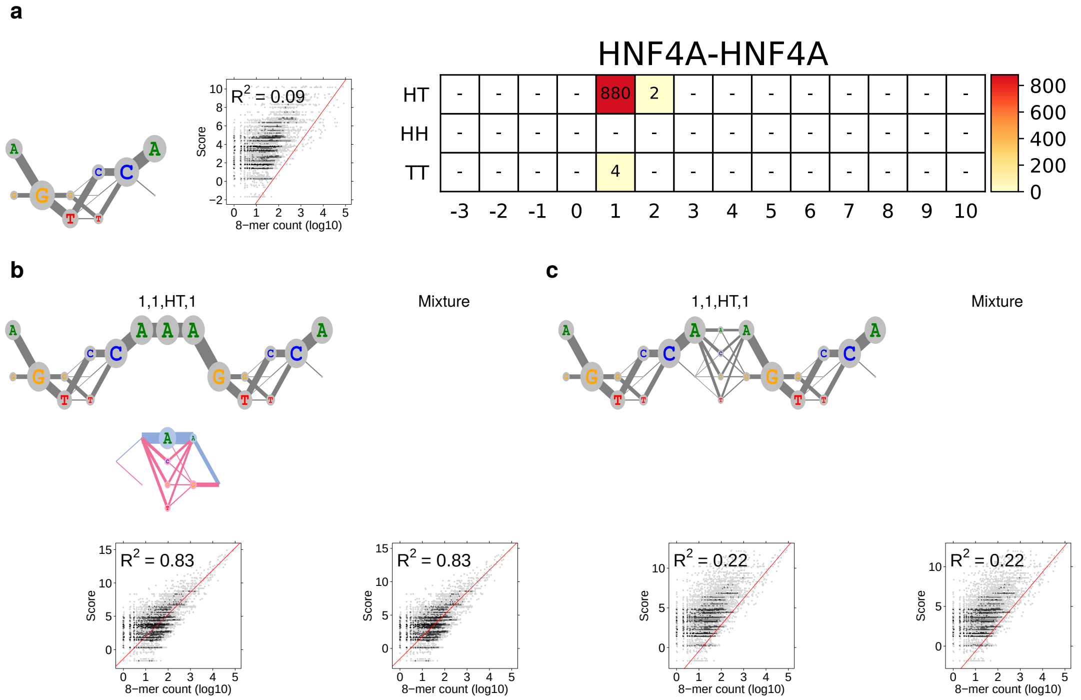

Figure S4: **Modularity analysis of HNF4A using one monomer, ADM case.** **(a)** Monomer ADM ($\lambda_1 = 0.017$) and the COB tables in units of integer multiples of 0.001, as learned from data by MODER2. **(b)** The 85% rule gives final mixture that includes only the dimeric ADM $\tau_{1,1,\mathrm{HT},1}$. Its deviation is depicted below the logo of the dimeric ADM. The mixture has the same correlation as its only component. Note that the COB table indicates a slight support for spacing 2. **(c)** Correlation analysis as in **b** but for the ADM $E_{1,1,\mathrm{HT},1}$ that is expected under the independence assumption. The R^2-values for the learned and expected ADMs differ remarkably, reflecting the large deviation between the learned and the expected ADMs. The expected model does not detect the `AAA` sequence connecting the half-sites. On the other hand, even the learned ADM does not explain the data well ($R^2 = 0.83$), because here the half-sites of the dimer are forced to be equal.

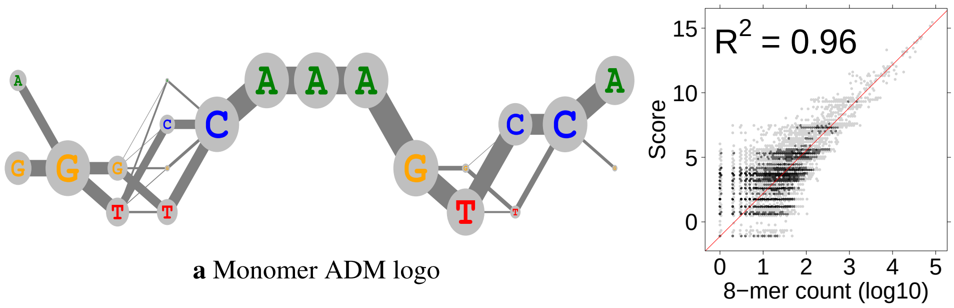

a Monomer ADM logo

b Monomer ADM correlation

Figure S5: **Modularity analysis of HNF4A using one monomer ADM, no dimers. (a)** Logo of the ADM model ($\lambda = 0.886$). **(b)** Correlation analysis. The single monomer ADM has the same correlation as the dimeric ADM of Fig. 3.

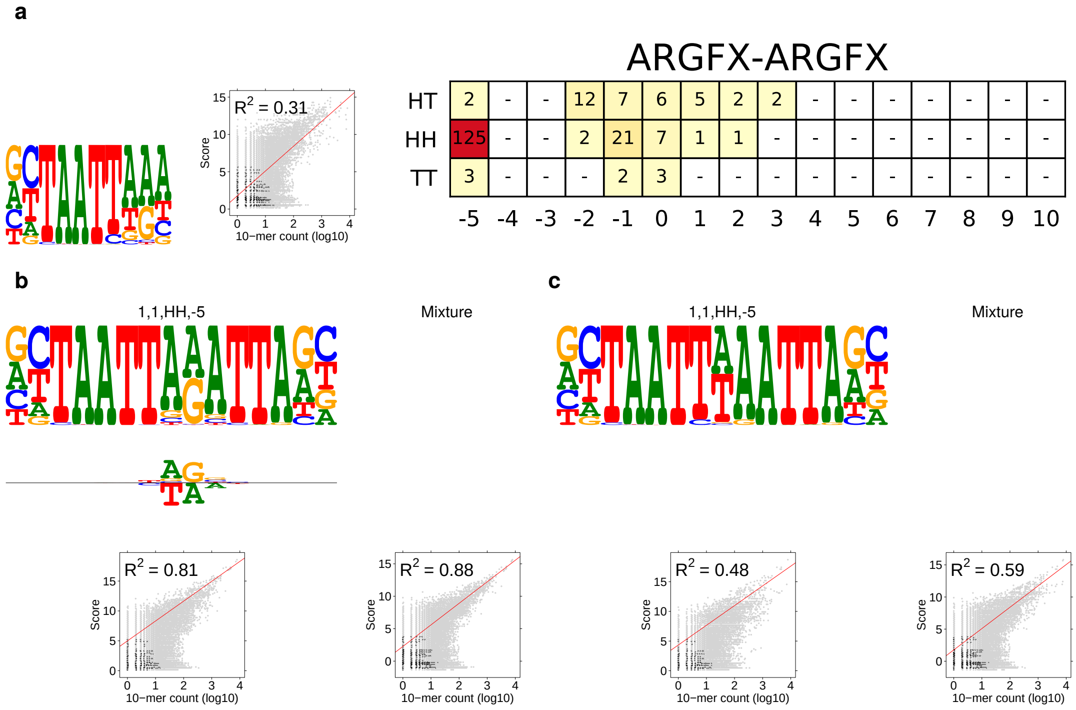

Figure S6: **Modularity analysis of ARGFX, PPM case.** As Fig. 4 but for PPMs. The mixing parameters of the mixture of **b** and **c** are $\lambda_1 = 0.205$ and $\lambda_{1,1,HH,-5} = 0.125$.

S6 Performance comparison of MODER2, BaMM, and InMoDe

	R^2	Median R^2	Time	Space
MODER2-order-0-monomeric	0.647	0.696	00:02:35	135.52 MiB
MODER2-order-0-dimeric	0.682	0.743	07:27:54	10.59 GiB
MODER2-order-1-monomeric	0.696	0.750	00:09:55	367.16 MiB
MODER2-order-1-dimeric	**0.738**	**0.800**	15:02:29	10.59 GiB
BaMM-order-1	0.693	0.743	00:14:43	1.04 GiB
BaMM-order-2	0.698	0.730	00:19:14	1.04 GiB
InMoDe	0.729	0.764	06:04:56	12.53 GiB

	Average R^2			
Domain	bHLH	bZIP	ETS	Homeodomain
MODER2-order-0-monomeric	0.632	0.652	0.707	0.639
MODER2-order-0-dimeric	0.636	0.683	0.717	0.694
MODER2-order-1-monomeric	0.682	0.751	0.741	0.676
MODER2-order-1-dimeric	0.686	0.768	0.773	**0.742**
BaMM-order-1	0.687	0.706	0.757	0.680
BaMM-order-2	0.709	0.701	**0.779**	0.677
InMoDe	**0.716**	**0.774**	0.738	0.718
	Median R^2			
Domain	bHLH	bZIP	ETS	Homeodomain
MODER2-order-0-monomeric	0.721	0.743	0.840	0.664
MODER2-order-0-dimeric	0.742	0.762	0.839	0.737
MODER2-order-1-monomeric	0.752	0.827	0.865	0.719
MODER2-order-1-dimeric	**0.775**	**0.850**	**0.885**	**0.792**
BaMM-order-1	0.739	0.775	0.747	0.725
BaMM-order-2	0.714	0.771	0.811	0.714
InMoDe	0.740	0.798	0.826	0.748

Table S4: Median R^2, average R^2, average running times, and memory requirements for the tested algorithms in total data and in families bHLH, bZIP, ETS, Homeodomain.

S7 Symbol definitions

a	nucleotide in the previous position
b	nucleotide in the current position
B_1, B_2	the set of positions in a sequence that are outside motif occurrences. That is, background positions.
c	additional nucleotide
d	distance between two occurrences.
D^+	independent dimer indices $\{k_1k_2od : d \geq \delta, k_1, k_2 \in M\}$
D^-	dependent dimer indices $D^- = \{k_1k_2od : d < \delta, k_1, k_2 \in M\}$
$E_{k_1k_2od}$	expected dimer model
f	helper function needed in defining "intersection-product" of ADM models
h	position in the motif belonging to range $1, \ldots, \ell$
H	height of a model: 4 for PPMs and 16 for ADMs
$\mathscr{H}_\rho(x)$	ρ-Hamming neighbourhood of k-mer x
$\mathscr{H}$	Hamming distance $\mathscr{H}(x, y)$ between two k-mers x and y
$I_{\ell_k}(i, j)$	$H \times \ell_k$ matrix-valued indicator function
$J_{\ell_k}(i, j)$	$H \times \ell_k$ matrix-valued indicator function for seed bias correction of the sample of sites defined by the Hamming radius.
$J'_{\ell_k}(i, j)$	$H \times \ell_k$ matrix-valued indicator function for handling only the seed bias of 0th degree of the sample of sites defined by the Hamming radius.
i	indexes through the set of sequences X.
j	starting position of a motif occurrence in a sequence
k	refers to a motif. If $k \in 1, \ldots, p$, it is a monomer. If $k = 0$, then it refers to background model. If $k = k_1k_2od$, then it refers to the corresponding dimer. Sometimes also the length of a k-mer.
L_i	length of ith sequence
ℓ	length of a model
M	monomer indices $M = \{1, \ldots, p\}$
n	number of sequences
o	orientation
p	number of monomer models
Q_X	counts of alphabet symbols in the full data X
R	Set of TF combinations, which we are interested in: $R \subset \{1, 2, \ldots, p\}^2$
r	number of mismatches between the prefix of a site and the seed, $0 \leq r \leq \rho - 1$
r'	number of mismatches between the suffix of a site and the seed, $0 \leq r' \leq \rho - 1$

S_{ik}	the set of all possible starting positions of a model θ_k in sequence X_i
S	IUPAC seed. Also a score.
s	seed
t	EM iteration: $t \in \{1,\ldots,\texttt{maxiter}\}$
X	The multiset of sequences: $X = (X_1, X_2, \ldots, X_n)$. Also a Markov chain.
Y	a Markov chain
Z	hidden variables that tell the starting positions of the models in the sequences. Also a Markov chain: intersection-product of two Markov chains X and Y
x, y	k-mers
W_k	weight or count matrix of model k
$W^{ab,h}$	an element of a count/weight matrix, also written as $W[ab,h]$
W	a product Markov chain
$\gamma^{b,h}(r')$	seed bias correction divisor
δ	minimum value for distance between motif occurrence, so that the occurrences are considered independent
Δ	range of allowed distances $[dmin, dmax]$
ε	EM convergence threshold
η	total model (θ, ψ, λ)
λ_k	fraction of sequences explained by model k
ψ	bridging segment
κ	deviation between the observed model and the expected model
ρ	Hamming radius
Σ	the alphabet $\{\mathrm{A}, \mathrm{C}, \mathrm{G}, \mathrm{T}\}$
θ_0	background model
θ_U	uniform background model: $\theta_U[b] = 1/4$ for all $b \in \Sigma$
θ_k	monomer model for $k \subset \{1, 2, \ldots, p\}$
$\tau_{k_1 k_2 od}$	dimeric model
Ω	the set of possible orientations
ω	$\omega(x)$ weigth/count of the k-mer x.
$\omega^{ab,h}(r)$	an uncorrected element of a count/weight matrix, also written as $\omega[ab,h,r]$

Bibliography

Bailey, T. L. and C. Elkan (1995). "The Value of Prior Knowledge in Discovering Motifs with MEME". In: *Proc. Third Internat. Conf. on Intelligent Systems for Molecular Biology,* AAAI Press, 21–29.

Bi, C. and P. K. Rogan (2004). "Bipartite pattern discovery by entropy minimization-based multiple local alignment". *Nucleic Acids Res.* 32, 4979–4991.

Dempster, A. P., N. M. Laird, and D. B. Rubin (1977). "Maximum likelihood from incomplete data via the EM algorithm". *Journal of the Royal Statistical Society, Series B* 39, 1–38.

Jolma, A., T. Kivioja, J. Toivonen, et al. (2010). "Multiplexed massively parallel SELEX for characterization of human transcription factor binding specificities". *Genome Res.* 20, 861–873.

Toivonen, J., J. Taipale, and E. Ukkonen (2017). "Seed-driven Learning of Position Probability Matrices from Large Sequence Sets". In: *17th International Workshop on Algorithms in Bioinformatics (WABI 2017).* Ed. by R. Schwartz and K. Reinert. Vol. 88. Leibniz International Proceedings in Informatics (LIPIcs). Dagstuhl, Germany: Schloss Dagstuhl–Leibniz-Zentrum fuer Informatik, 25:1–25:13.
